# Performance of risk prediction models for pancreatic cancer in patients with new-onset diabetes: a systematic review and meta-analysis

**DOI:** 10.3389/fonc.2026.1868121

**Published:** 2026-08-18

**Authors:** Dan Li, Nannan Zhang, Rui Li, Yaqi Tian, Gao Le, Guoqing Zhang

**Affiliations:** Senior Department of Oncology, Chinese People’s Liberation Army General Hospital, Beijing, China

**Keywords:** ENDPAC, meta-analysis, new-onset diabetes, pancreatic cancer, risk prediction model, risk stratification

## Abstract

**Background:**

New-onset diabetes (NOD) can precede the clinical diagnosis of pancreatic ductal adenocarcinoma (PDAC) and may indicate occult or preclinical disease. However, identifying the small subgroup of patients with NOD who are at sufficiently high risk to justify further evaluation remains challenging. This systematic review and meta-analysis evaluated the performance of PDAC risk prediction models in patients with NOD and summarized their reported discriminative ability.

**Methods:**

We systematically searched PubMed, Embase, and Web of Science from inception to March 16, 2026. Eligible studies developed or validated PDAC risk prediction models in patients with NOD. Risk of bias was assessed using the Prediction model Risk Of Bias Assessment Tool (PROBAST). Concordance indices (C-indices) were pooled using a random-effects model. For validation cohorts without reported variance estimates, standard errors were approximated using the Hanley-McNeil method. Subgroup analyses, meta-regression, and sensitivity analyses were performed to explore heterogeneity and assess the robustness of the findings.

**Results:**

Fourteen studies comprising 17 prediction models were included, and 14 independent validation cohort estimates were available for quantitative synthesis. Most models had a high risk of bias in the analysis domain. The pooled C-index was 0.78 (95% confidence interval, 0.75-0.82), with a 95% prediction interval of 0.68-0.86 and substantial heterogeneity (I² = 95.0%). This estimate should be interpreted as a descriptive summary of reported discrimination across heterogeneous validation cohorts rather than as a directly generalizable estimate of clinical performance. Exploratory subgroup analyses suggested higher discrimination in prospective validation cohorts and in models with 2-year prediction windows than in models with 3-year or other/unspecified prediction windows, although several subgroups included few validation cohorts and residual heterogeneity remained substantial. Models evaluated using internal validation showed numerically higher discrimination than those evaluated using independent external validation, suggesting potential optimism. Older age, unintentional weight loss, rapid glycemic deterioration, and proton pump inhibitor use were recurrent clinical warning signals.

**Conclusion:**

Existing PDAC risk prediction models for patients with NOD show moderate discrimination. However, substantial heterogeneity, limited independent external validation, incomplete calibration reporting, and inconsistent assessment of clinical utility constrain their immediate clinical translation. These models should be interpreted as tools for temporal risk stratification rather than as evidence of a causal relationship between NOD and PDAC onset. Future studies should standardize NOD definitions, improve calibration and interpretability reporting, and prioritize prospective external validation and clinical impact studies before implementation in screening pathways.

**Systematic Review Registration:**

https://www.crd.york.ac.uk/PROSPERO, identifier CRD420261360099.

## 1 Introduction

Pancreatic ductal adenocarcinoma (PDAC) is among the most lethal malignancies worldwide, with a 5-year survival rate below 12%. It is projected to become the second leading cause of cancer-related death by 2030 (1). This high mortality is largely attributable to nonspecific early symptoms and the lack of validated early detection strategies (2). Because the incidence of PDAC in the general population is low, population-wide imaging screening is not cost-effective (3). Moreover, guideline-recommended surveillance based on family history or inherited genetic mutations captures fewer than 10% of patients in real-world practice (4). Therefore, accessible clinical warning signals are needed to enrich populations who may benefit from targeted evaluation.

Recent prospective cohort studies have shown that glycemically defined NOD in individuals older than 50 years is associated with a 6- to 8-fold higher probability of PDAC diagnosis within 3 years compared with the general population (5). Basic and epidemiological studies have described a complex bidirectional relationship between PDAC and diabetes (6). Long-standing type 2 diabetes mellitus (T2DM) is an established risk factor for PDAC. In contrast, NOD occurring shortly before cancer diagnosis may reflect reverse causality, whereby occult or preclinical PDAC contributes to metabolic deterioration before the tumor becomes clinically apparent. Approximately 50% of patients with PDAC develop new-onset hyperglycemia or diabetes within 3 years before diagnosis, suggesting that NOD may serve as a clinical marker of occult PDAC rather than evidence that diabetes causes cancer onset during this short interval (7). However, distinguishing tumor-associated pancreatogenic diabetes from ordinary T2DM related to metabolic syndrome remains a major clinical challenge (8).

International oncology societies do not currently recommend costly screening for all patients with NOD (9). Risk prediction models may therefore provide a practical strategy for enriching the NOD population to identify higher-risk individuals who could be considered for targeted surveillance after appropriate validation. Several clinical, biochemical, and genetic prediction models have been developed in recent years. However, their performance, applicability, and methodological quality have not been systematically assessed using quantitative synthesis. The PROBAST tool provides a standardized framework for assessing risk of bias in prediction model studies (10, 11). To the best of our knowledge, this study provides a comprehensive systematic review and meta-analysis focused on PDAC risk prediction models in the NOD population. We aimed to summarize reported discrimination, methodological quality, calibration reporting, and clinical applicability, and to inform future model development, validation, and evaluation of clinical utility (12, 13).

## 2 Materials and methods

### 2.1 Study registration

This systematic review and meta-analysis was conducted in accordance with the Preferred Reporting Items for Systematic Reviews and Meta-Analyses (PRISMA) 2020 guidelines. The protocol was prospectively registered in the International Prospective Register of Systematic Reviews (PROSPERO; registration number: CRD420261360099) to ensure transparency and methodological rigor.

### 2.2 Search strategy

We systematically searched PubMed, Embase, and Web of Science from inception to March 16, 2026. The search strategy combined Medical Subject Headings (MeSH)/Emtree terms and free-text terms for three core concepts: (1) pancreatic cancer and related neoplasms; (2) diabetes, including new-onset diabetes; and (3) prediction models, nomograms, scoring systems, and machine-learning or deep-learning algorithms. Detailed database-specific search strategies are provided in the **Supplementary Material**. Two independent reviewers also manually screened the reference lists of included studies and related systematic reviews to identify additional eligible records.

### 2.3 Inclusion and exclusion criteria

Inclusion criteria: Studies were eligible if they met all of the following criteria: (1) study population: adult patients with NOD, defined as diabetes diagnosed within ≤3 years; (2) prediction model: development and/or validation of a PDAC risk prediction model, including internal validation, internal-external validation, or independent external validation; (3) outcomes: reporting of the concordance index (C-index) or area under the receiver operating characteristic curve (AUC), together with variance data or sufficient information to estimate variance; and (4) study design: original observational studies, including cohort or case-control studies.

Exclusion criteria: Studies were excluded if they (1) focused exclusively on hereditary PDAC; (2) did not report model performance metrics; (3) were basic science studies; (4) were conference abstracts; or (5) were reviews.

### 2.4 Study selection and data extraction

Two reviewers independently screened titles and abstracts and then assessed full texts. Disagreements were resolved by discussion or, when necessary, by consultation with a third reviewer. We extracted the following information from each included study: first author, publication year, country, study design, data source, sample size, number of PDAC events, prediction window, model type, predictors, and the C-index or AUC with the corresponding 95% confidence interval (CI).

### 2.5 Quality evaluation

We used PROBAST to assess risk of bias and applicability in the included studies (11). PROBAST evaluates four domains: participants, predictors, outcome, and analysis. If any domain was rated as high risk, the overall risk of bias was classified as high. Two reviewers independently completed the assessments.

### 2.6 Validation classification

Validation strategies were classified as internal validation, internal-external validation, or independent external validation. Internal validation included bootstrap resampling, cross-validation, split-sample validation, or validation within the same source population. Independent external validation was defined as validation in an independent population differing by institution, geography, time period, or data source. Internal-external validation referred to validation across different centers, regions, or time periods within a shared study framework. When reported, the type of validation was extracted and classified accordingly (11, 29).

### 2.7 Statistical analysis and data synthesis

The unit of analysis for quantitative synthesis was the independent validation cohort rather than the individual model or the original study. When multiple models were reported within the same validation cohort, we selected the model with the most complete validation information or the model recommended by the original authors for clinical use to avoid double-counting participants. When an original study reported multiple truly independent validation cohorts, each validation cohort was treated as a separate analytical unit. Potential overlap in source populations was assessed according to data source, study period, geographical region, and cohort characteristics. We pooled C-indices and their 95% CIs from independent validation cohorts to provide a descriptive summary of reported discriminative performance. Given the anticipated clinical and methodological heterogeneity across validation cohorts, the pooled C-index was not intended to represent a directly transportable estimate for clinical implementation in a specific setting. For validation cohorts in which the original publication did not report the 95% CI or variance of the C-index or AUC, we estimated the standard error using the Hanley-McNeil method (14). For binary-outcome discrimination measures, the C-index and AUC were treated as equivalent discrimination metrics for variance approximation. Hanley-McNeil imputation was not applied to time-to-event C-indices when variance estimates were reported. This approach assumes independent observations and an approximately normal sampling distribution for the AUC or C-index estimate. We performed a sensitivity analysis excluding validation cohorts with imputed standard errors to assess whether variance imputation influenced the primary pooled C-index. Random-effects models were used for all pooled analyses to account for anticipated clinical and methodological heterogeneity among validation cohorts. Heterogeneity was quantified using the I² statistic. To explore potential sources of heterogeneity, we conducted predefined subgroup analyses and univariable meta-regression. All statistical analyses were performed using R software (version 4.5.2).

## 3 Results

### 3.1 Literature selection

**Figure 1:**
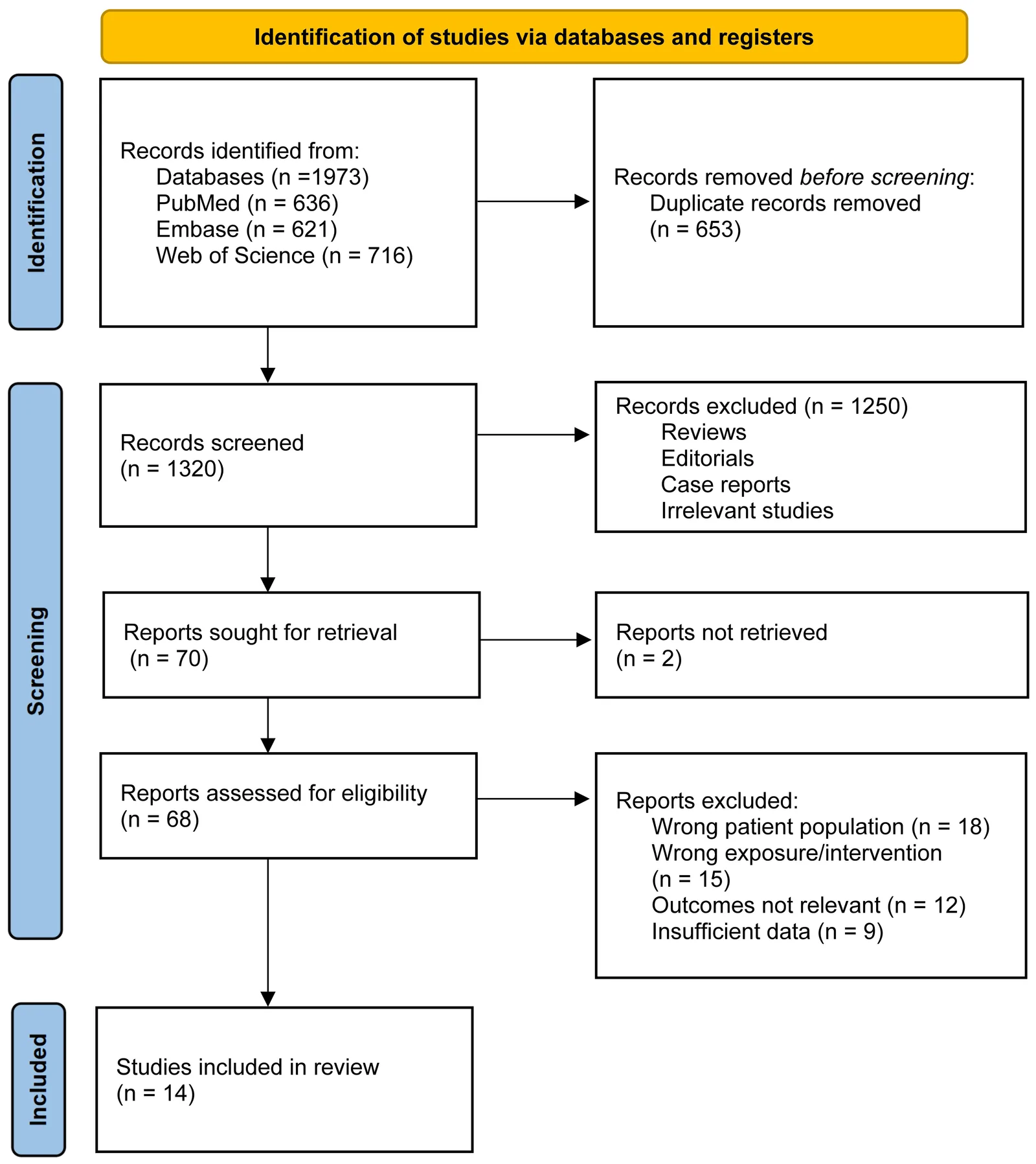
PRISMA flow diagram illustrating the literature search and study selection process.

The initial search retrieved 1,973 records. After duplicates were removed, 1,320 unique records remained. Following independent title/abstract screening and full-text review, 14 studies were included, comprising 17 prediction models and 14 independent validation cohorts available for quantitative synthesis. For studies that reported multiple models in the same population, one representative model estimate was selected for quantitative synthesis to avoid double-counting. The study selection process is shown in **Figure 1**.

### 3.2 Characteristics of included studies

The 14 included studies (15–28) were published between 2017 and 2025, with total sample sizes ranging from 1,561 to 1,358,634 and PDAC events ranging from 16 to 3,092. Studies were conducted mainly in Europe, North America, and Asia. The included studies used logistic regression, Cox proportional hazards regression, the ENDPAC score, Random Forest, and XGBoost algorithms. Most models used routinely available clinical indicators as predictors, whereas some incorporated genetic markers. Nine studies performed internal validation only, one used internal-external validation, and four conducted independent external validation, mainly of the ENDPAC model (**Table 1**).

**Table 1:** Baseline characteristics of the included studies.

Study ID	Country	Study design	Data source	Sample size (n)	Events (n)	Event rate (%)	Target population	Prediction window (years)
Ali 2024 (15)	Australia	Retrospective	Admin Database	99,687	602	0.60	Females with NODM (≥50 y)	3
Boursi 2017 (16)	UK	Retrospective	THIN	109,385	390	0.36	Patients with NODM	3
Boursi 2022 (IL)* (17)	Israel	Retrospective	MHS	5,408	59	1.09	NODM (≥50 y)	3
Boursi 2022 (UK) (18)	UK	Retrospective	THIN	138,232	245	0.18	Newly diagnosed IFG (≥35 y)	3
Chen 2021* (19)	USA	Retrospective	KPSC	13,947	99	0.71	NODM (50–84 y)	3
Cichosz 2024 (20)	Denmark	Retrospective matched case-control	National Registry	1,432	716	50.00	NODM (≥50 y)	3
Clift 2024 (21)	UK	Retrospective	QResearch	253,766	767	0.30	Newly diagnosed T2DM (30–85 y)	2
Hsieh 2018 (22)	Taiwan, China	Retrospective	NHIRD	1,358,634	3,092	0.23	Newly diagnosed T2DM	Other/Unspecified
Khan 2021* (23)	USA	Retrospective	TriNetX	6,302	48	0.76	NODM (≥50 y)	4
Khan S 2024 (24)	USA	Retrospective	TriNetX	81,213	380	0.47	NODM (≥50 y)	3
Sharma 2018* (25)	USA	Retrospective	REP	1,561	16	1.02	NODM (≥50 y)	3
Sharma 2022 (26)	UK	Prospective cohort-derived enriched subgroup	UK Biobank	205	135	65.85	NODM Subgroup	2
Sun 2024 (27)	UK	Prospective	UK Biobank	12,735	100	0.79	NODM	2
Zhou 2025 (28)	UK	Prospective	UK Biobank	15,063	238	1.58	NODM	3

IFG, impaired fasting glucose; KPSC, Kaiser Permanente Southern California; MHS, Maccabi Healthcare Services; NHIRD, National Health Insurance Research Database; NODM, new-onset diabetes mellitus; NR, not reported; REP, Rochester Epidemiology Project; T2DM, type 2 diabetes mellitus; THIN, The Health Improvement Network.

All included studies, except Sharma 2018, utilized multicenter designs. The disproportionately high event rates observed in Cichosz 2024 (50.00%) and Sharma 2022 (65.85%) are attributable to a matched case-control design or a cohort-derived enriched subgroup, and therefore do not reflect the absolute risk in general real-world NOD cohorts.

*Indicates validation cohorts for which the original publications did not report variance data. For these four validation cohorts, standard errors and 95% CIs were estimated using the Hanley-McNeil formula to enable inclusion in the quantitative synthesis. Detailed estimation parameters are provided in **Supplementary Table 1**.

### 3.3 Model characteristics and validation strategies

Most models were evaluated using internal validation, whereas only one study used internal-external validation and independent external validation was limited. Specifically, nine studies performed internal validation only, one used an internal-external validation framework, and four conducted independent external validation, mainly for the ENDPAC model. Because internal validation can produce optimistic performance estimates, models evaluated using independent external validation provide more informative evidence for real-world applicability (11, 29, 30). Reporting of machine-learning interpretability methods, such as SHAP values or permutation-based feature importance, was limited.

### 3.4 Methodological quality evaluation (PROBAST)

**Figure 2:**
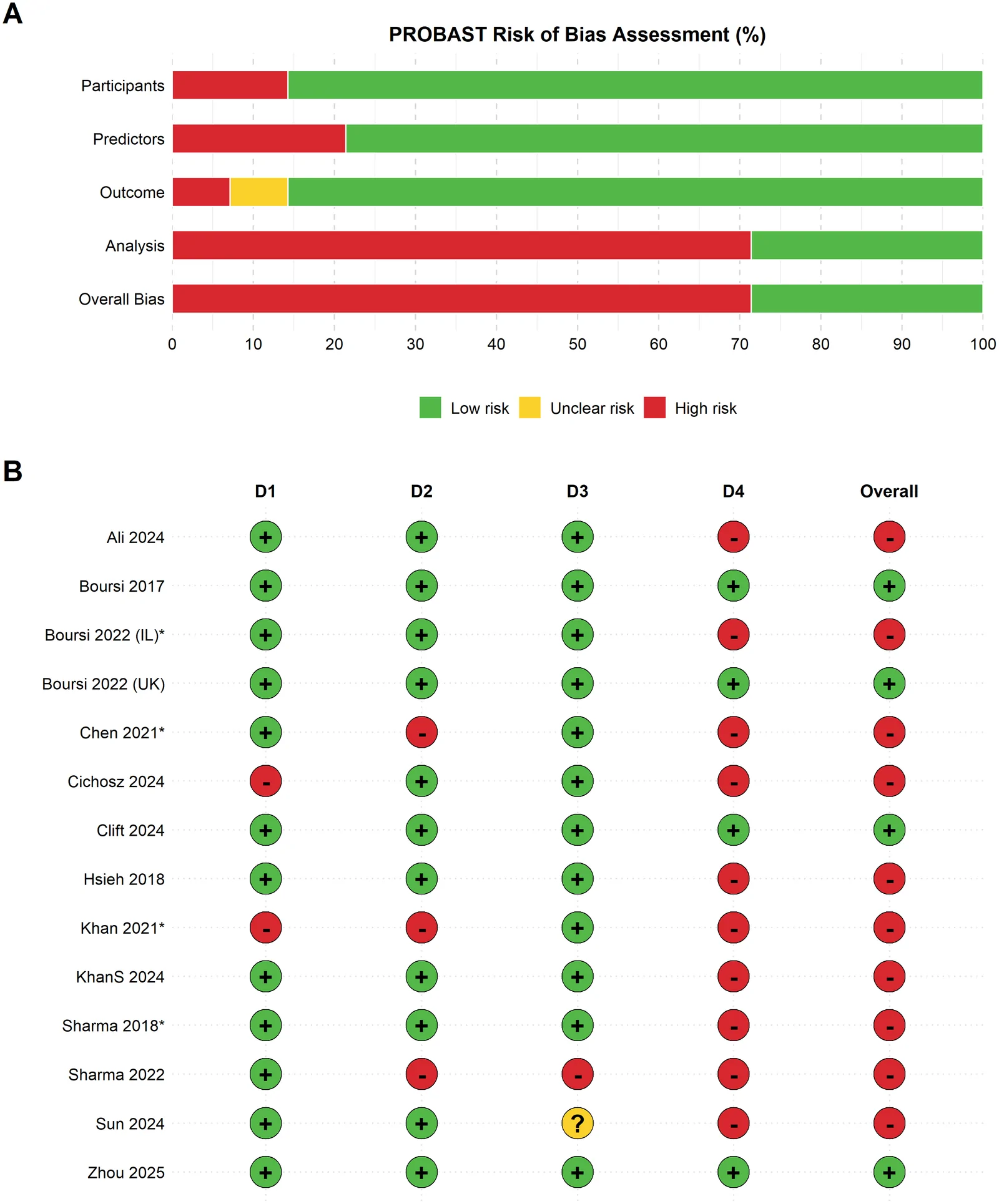
Risk of bias assessment of the included studies using the PROBAST tool. **(A)** Stacked bar chart depicting the overall percentage of studies with low, unclear, or high risk of bias across the four domains and the overall assessment. **(B)** Traffic light plot displaying the domain-level and overall risk of bias for each individual study. D1: Participants; D2: Predictors; D3: Outcome; D4: Analysis. Green circles with “+” indicate a low risk of bias; yellow circles with “?” indicate an unclear risk of bias; red circles with “-” indicate a high risk of bias. Abbreviations: PROBAST, Prediction model Risk Of Bias Assessment Tool.

Using PROBAST, 10 studies (71.4%) were judged to have an overall high risk of bias (**Supplementary Table 2**). High risk of bias arose almost exclusively from the analysis domain. Common methodological limitations included a critically low events-per-variable ratio, inappropriate handling of missing data, lack of internal validation or calibration reporting, and failure to apply penalization techniques to reduce overfitting. In contrast, risk of bias in the participants, predictors, and outcome domains was generally low (**Figure 2**).

### 3.5 Meta-analysis of model discrimination

#### 3.5.1 Overall pooled effect and missing variance imputation

**Figure 3:**
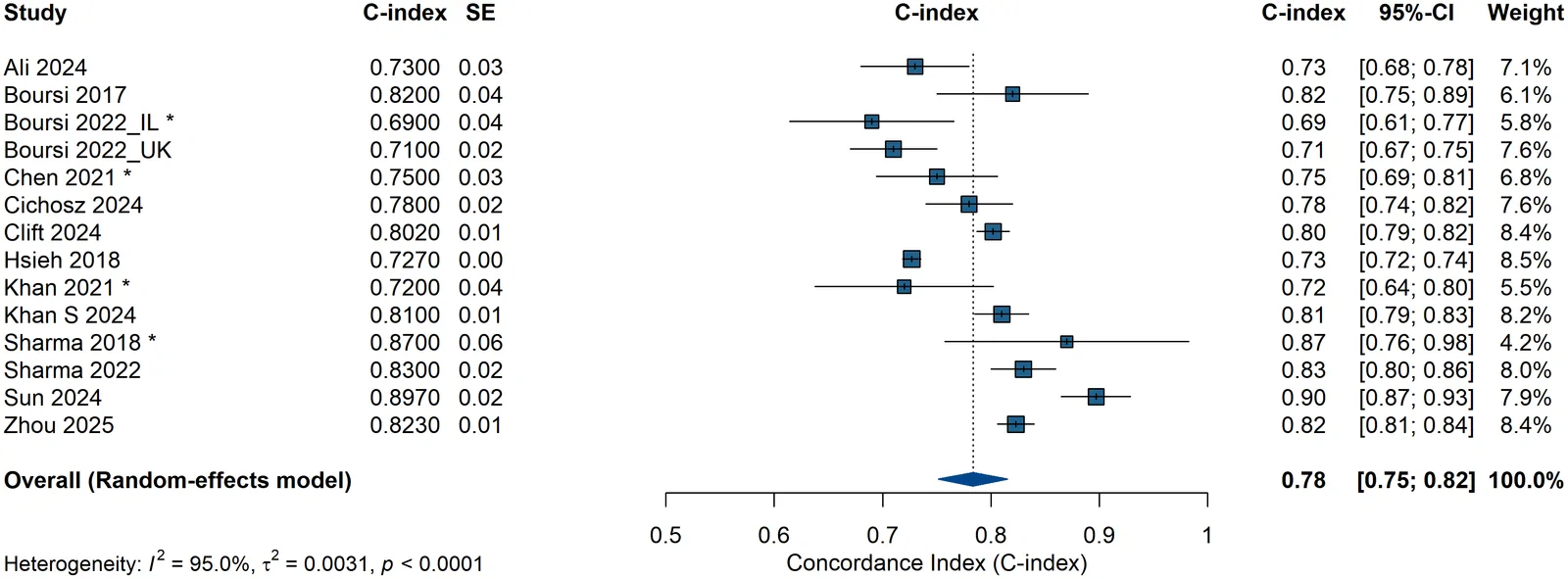
Forest plot of pooled concordance index (C-index) estimates from validation cohorts for pancreatic cancer prediction models in patients with NOD. Each square represents the C-index estimate from an individual validation cohort, and the horizontal line through each square indicates the corresponding 95% confidence interval (CI). The size of each square is proportional to the inverse-variance weight assigned in the random-effects meta-analysis. The diamond represents the pooled C-index and its 95% CI. The included estimates were derived from internal validation, internal-external validation, or independent external validation cohorts, which have different evidentiary value. Estimates from internal validation may be more optimistic than estimates from independent external validation. Therefore, the pooled estimate should be interpreted as a descriptive summary of reported discrimination across validation settings rather than as a directly transportable performance estimate. CI, confidence interval; C-index, concordance index; NOD, new-onset diabetes; PDAC, pancreatic ductal adenocarcinoma. Asterisks after cohort labels indicate validation cohorts for which standard errors were imputed using the Hanley-McNeil method. The vertical dotted reference line indicates the pooled C-index estimate.

We pooled C-indices across 14 independent validation cohorts using a random-effects model. The representative validation cohort estimates included in the quantitative synthesis are summarized in **Table 2**. The overall pooled C-index was 0.78 (95% CI: 0.75-0.82), with substantial heterogeneity (I² = 95.0%, P < 0.001) and a 95% prediction interval of 0.68-0.86 (**Figure 3**). This high heterogeneity likely reflected differences in NOD definitions, age thresholds, prediction windows, predictor sets, data sources, model algorithms, and validation strategies. Therefore, the pooled C-index should be interpreted as a descriptive summary of reported discrimination across heterogeneous validation cohorts rather than as a directly generalizable estimate of clinical performance. For the four independent external validation cohorts without published variance data (Boursi 2022_IL, Chen 2021, Khan 2021, and Sharma 2018), standard errors (SEs) were estimated using the Hanley-McNeil formula. Although PDAC event counts ranged from 16 to 99 in these retrospective validation cohorts, the estimated SEs remained within a plausible range (0.028-0.057), allowing these cohorts to be included in the descriptive quantitative synthesis (**Supplementary Table 1**). The influence of these imputed variance estimates was further assessed in sensitivity analyses.

**Table 2:** Predictive performance and validation details.

Study ID	Model evaluated	Model type	Validation method	C-index (95% CI)	Sensitivity	Specificity
Ali 2024 (15)	Unnamed	Logistic Regression	Internal	0.73 (0.68–0.78)	0.69	0.69
Boursi 2017 (16)	Unnamed	Logistic Regression	Internal	0.82 (0.75–0.89)	0.45	0.94
Boursi 2022(IL)* (17)	ENDPAC	Logistic Regression	External	0.69 (0.61–0.77)	0.54	0.77
Boursi 2022 (UK) (18)	Unnamed	Logistic Regression	Internal	0.71 (0.67–0.75)	0.67	0.55
Chen 2021* (19)	ENDPAC	Logistic Regression	External	0.75 (0.69–0.81)	0.63	0.79
Cichosz 2024 (20)	Random Forest	Machine Learning	Internal	0.78 (0.75–0.83)	NA	NA
Clift 2024 (21)	QPancreasD	Cox Regression	Internal-external	0.80 (0.79–0.82)	0.04	0.99
Hsieh 2018 (22)	Unnamed	Logistic Regression	Internal	0.73 (0.72–0.74)	1.00	NA
Khan 2021* (23)	ENDPAC	Logistic Regression	External	0.72 (0.64–0.80)	0.56	0.75
Khan S 2024 (24)	XGBoost	Machine Learning	Internal	0.81 (0.78–0.83)	0.75	0.70
Sharma 2018* (25)	ENDPAC	Logistic Regression	External	0.87 (0.76–0.98)	0.80	0.80
Sharma 2022 (26)	Clinical + Genetic	Logistic Regression	Internal	0.83 (0.80–0.86)	0.29	0.91
Sun 2024 (27)	Clinical + Genetic	Logistic Regression	Internal	0.90 (0.87–0.93)	0.76	0.88
Zhou 2025 (28)	Model 2	Cox Regression	Internal	0.82 (0.81–0.84)	0.08	0.99

CI, confidence interval; NA, not available.

Each row corresponds to the representative estimate entering the quantitative synthesis, not every model reported in the original study.

Indicates validation cohorts for which the original publications did not report variance data. For these four validation cohorts, standard errors and 95% CIs were estimated using the Hanley-McNeil formula to enable inclusion in the quantitative synthesis (see **Supplementary Table 1** for detailed estimation parameters).

Validation type was considered during interpretation because internal validation is more prone to optimism than independent external validation.

#### 3.5.2 Subgroup analyses and heterogeneity exploration

To explore the substantial heterogeneity observed in the overall analysis (I² = 95.0%), we conducted subgroup analyses by study design, prediction window, validation method, methodological quality, and clinical characteristics. Because several subgroups included only one to three validation cohorts, all subgroup findings should be interpreted as exploratory. Detailed results are provided in **Table 3**. In the subgroup analyses, k denotes the number of independent validation cohorts included in each subgroup, whereas the numbers in parentheses after k indicate the corresponding reference numbers for those validation cohort estimates.

**Table 3:** Subgroup analyses of pooled C-index for new-onset diabetes prediction models.

Subgroups/characteristics	No. of validation cohorts, k (refs.)	Pooled C-index (95% CI)	I^2^(%)	P for heterogeneity	χ² value	P for subgroup difference
Study design					9.91	0.002
Retrospective	11 (15-25)	0.76 (0.73-0.79)	92.1	<0.001		
Prospective	3 (26-28)	0.85 (0.80-0.89)	89.4	<0.001		
Prediction window					22.32	<0.001
3 Years	9 (15-20,24, 25, 28)	0.77 (0.74-0.81)	83.9	<0.001		
2 Years	3 (21, 26, 27)	0.84 (0.79-0.90)	93.6	<0.001		
Other/Unspecified	2 (22, 23)	0.73 (0.72-0.74)	0.0	0.865		
Target population					1.40	0.236
Ultra high-risk (Age ≥ 50)	7 (15, 17, 19, 20, 23-25)	0.76 (0.73-0.80)	72.9	0.001		
General NOD population	7 (16, 18, 21, 22, 26-28)	0.80 (0.75-0.85)	97.7	<0.001		
Validation method					2.78	0.249
Internal validation only	9 (15-16, 18, 20, 22, 24, 26-28)	0.79 (0.75-0.83)	96.6	<0.001		
Internal-external validation	1 (21)	0.80 (0.79-0.82)	NA	NA		
Independent external validation	4 (17, 19, 23, 25)	0.75 (0.69-0.81)	57.9	0.068		
Model algorithm type					2.45	0.294
Logistic regression-based	10 (15, 16, 17, 18, 19, 22, 23, 25, 26, 27)	0.77 (0.73-0.82)	94.0	<0.001		
Machine learning	2 (20, 24)	0.80 (0.77-0.83)	35.6	0.213		
Cox regression	2 (21, 28)	0.81 (0.79-0.83)	69.7	0.069		
Geographical region					5.02	0.171
Oceania	1 (15)	0.73 (0.68-0.78)	–	–		
Europe	6 (16, 18, 20, 21, 26, 27)	0.81 (0.76-0.86)	91.8	<0.001		
Asia	3 (17, 22, 28)	0.75 (0.67-0.83)	98.4	<0.001		
North America	4 (19, 23-25)	0.78 (0.73-0.84)	66.7	0.029		
Data source type					4.17	0.124
National administrative registry	3 (15, 20, 22)	0.74 (0.71-0.78)	69.0	0.040		
Single database or cohort	9 (16-19, 21, 25-28)	0.80 (0.76-0.84)	89.3	<0.001		
Federated clinical network	2 (23, 24)	0.77 (0.69-0.86)	78.1	0.032		
PROBAST risk of bias					0.05	0.832
High risk of bias	10 (15, 17, 19, 20, 22-27)	0.78 (0.74-0.82)	95.3	<0.001		
Low risk of bias	4 (16, 18, 21, 28)	0.79 (0.74-0.84)	89.1	<0.001		

CI, confidence interval; NA, not applicable; NOD, new-onset diabetes; PROBAST, Prediction model Risk Of Bias Assessment Tool.

Subgroup analyses were based on independent validation cohorts, which were the unit of analysis for quantitative synthesis. The value of k represents the number of independent validation cohorts included in each subgroup, not the reference numbers. Numbers in parentheses after k indicate the corresponding reference numbers for the validation cohort estimates included in that subgroup. Pooled C-index estimates and 95% confidence intervals were calculated using a random-effects model. P values for subgroup differences were derived from Cochran’s Q test for between-subgroup heterogeneity. Heterogeneity within subgroups was assessed using the I² statistic where applicable. Because several subgroups contained only one to three validation cohorts and residual heterogeneity remained substantial, all subgroup findings should be interpreted as exploratory. For subgroups with a single validation cohort, the cohort-specific C-index and 95% confidence interval are presented, and within-subgroup heterogeneity could not be estimated. Validation method was classified as internal validation only, internal-external validation, and independent external validation because these approaches have different evidentiary value. In the model algorithm type subgroup, logistic regression-based models include both internally developed logistic regression models and independent external validation cohorts of the ENDPAC score, as ENDPAC was originally derived using logistic regression. The three ENDPAC independent external validation cohorts, Refs. 17, 19, and 23, were therefore classified under logistic regression-based models rather than as a separate algorithm category. Forest plots for subgroup analyses are provided in **Supplementary Figures 1-8**, and the sensitivity analysis excluding validation cohorts with imputed variance estimates is provided in **Supplementary Figure 9**. CI, confidence interval; C-index, concordance index; ENDPAC, Enriching New-Onset Diabetes for Pancreatic Cancer; I², inconsistency statistic; k, number of independent validation cohorts; RoB, risk of bias.

Exploratory subgroup analysis by study design suggested that prospective validation cohorts [k = 3 (26–28)] had higher pooled C-indices (0.85, 95% CI: 0.80-0.89; I² = 89.4%) than retrospective validation cohorts [k = 11 (15–25); pooled C-index = 0.76, 95% CI: 0.73-0.79; I² = 92.1%]. This subgroup difference was statistically significant (P = 0.002).

Exploratory subgroup analysis suggested that prediction window may influence reported model performance (P < 0.001). Models with a 2-year prediction window [k = 3 (21, 26, 27)] showed numerically higher discrimination (pooled C-index = 0.84, 95% CI: 0.79-0.90) than models with a 3-year prediction window [k = 9 (15–20, 24, 25, 28); pooled C-index = 0.77, 95% CI: 0.74-0.81] or other/unspecified prediction windows [k = 2 (22, 23); pooled C-index = 0.73, 95% CI: 0.72-0.74]. This pattern may indicate that 2-year prediction windows better capture metabolic changes occurring closer to the diagnosis of occult or preclinical PDAC. However, because only a limited number of validation cohorts contributed to this subgroup and residual heterogeneity remained substantial, this finding should be interpreted cautiously. The observed pattern reflects temporal enrichment of occult or preclinical PDAC-related metabolic signals rather than evidence that NOD causes PDAC onset.

Stratification by validation method showed that models evaluated by internal validation only [k = 9 (15, 16, 18, 20, 22, 24, 26–28)] yielded a pooled C-index of 0.79 (95% CI: 0.75-0.83; I² = 96.5%), whereas models evaluated by independent external validation [k = 4 (17, 19, 23, 25)] yielded a pooled C-index of 0.75 (95% CI: 0.69-0.81; I² = 57.9%). One study using internal-external validation [k = 1 (21)] reported a C-index of 0.80 (95% CI: 0.79-0.82). The subgroup difference was not statistically significant (χ² = 2.78, P = 0.249). Although models evaluated by internal validation only showed numerically higher discrimination than those evaluated by independent external validation, this finding should be interpreted cautiously because internal validation may produce optimistic estimates and several validation subgroups contained few validation cohorts. Therefore, independent external validation provides more informative evidence for assessing potential real-world applicability.

Methodological quality, as assessed by PROBAST, was not significantly associated with model discrimination (P = 0.832). Models classified as having a low risk of bias [k = 4 (16, 18, 21, 28)] yielded a pooled C-index of 0.79 (95% CI: 0.74-0.84), which was consistent with models rated as having a high risk of bias [k = 10 (15, 17, 19, 20, 22–27); pooled C-index = 0.78, 95% CI: 0.74-0.82].

Subgroup analysis by target population showed no significant difference in model discrimination (P = 0.236). Models developed or validated in general NOD cohorts [k = 7 (16, 18, 21, 22, 26–28)] yielded a pooled C-index of 0.80 (95% CI: 0.75-0.85). Models restricted to individuals aged ≥50 years or other high-risk NOD populations [k = 7 (15, 17, 19, 20, 23–25)] yielded a C-index of 0.76 (95% CI: 0.73-0.80).

Exploratory subgroup analysis by model algorithm type did not show statistically significant differences (P = 0.294). Logistic regression-based models [k = 10 (15–19, 22, 23, 25–27)] yielded a pooled C-index of 0.77 (95% CI: 0.73-0.82; I² = 94.0%), whereas Cox regression models [k = 2 (21, 28)] and machine-learning models [k = 2 (20, 24)] yielded pooled C-indices of 0.81 (95% CI: 0.79-0.83; I² = 69.7%) and 0.80 (95% CI: 0.77-0.83; I² = 35.6%), respectively.

No statistically significant subgroup difference was observed by geographical region (P = 0.170). Pooled C-indices were 0.81 for European cohorts [k = 6 (16, 18, 20, 21, 26, 27)], 0.78 for North American cohorts [k = 4 (19, 23–25)], 0.75 for Asian cohorts [k = 3 (17, 22, 28)], and 0.73 for the Oceanian cohort [k = 1 (15)].

Data source type was not significantly associated with discrimination (P = 0.124). Models derived from single databases or cohorts [k = 9 (16–19, 21, 25–28)] achieved a C-index of 0.80. Models based on federated networks [k = 2 (23, 24)] and national registries [k = 3 (15, 20, 22)] yielded C-indices of 0.77 and 0.74, respectively.

Substantial heterogeneity (I² > 70%) persisted within most strata despite subgrouping, suggesting that study-level clinical variation and differences in data source structure continued to contribute to the observed variability.

#### 3.5.3 Meta-regression suggests potential optimism bias and spectrum bias

**Figure 4:**
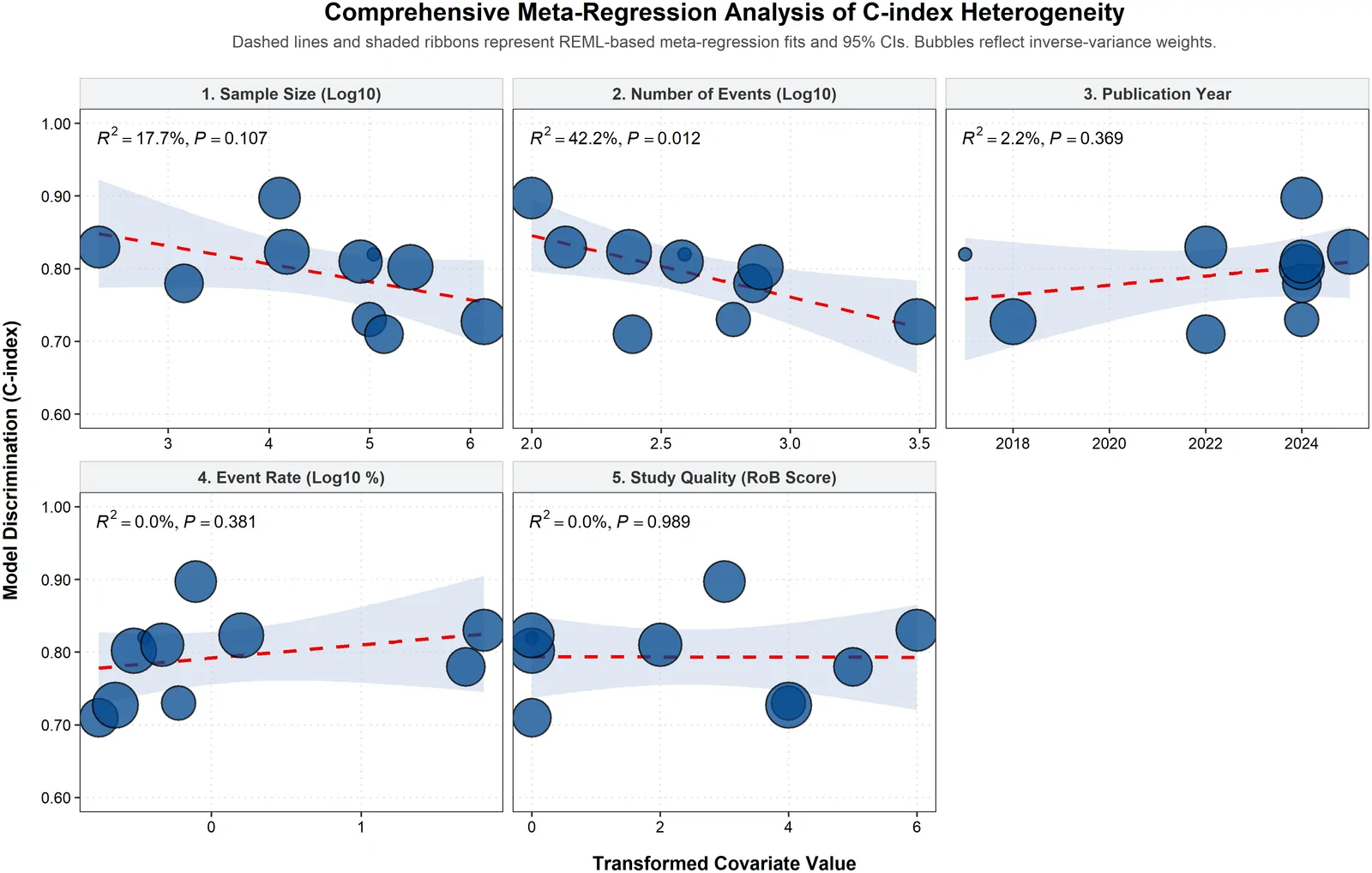
Univariable meta-regression analysis of potential sources of heterogeneity for model discrimination. The multi-panel bubble plots illustrate the relationship between reported discrimination and five study-level covariates: (1) log10-transformed sample size, (2) log10-transformed absolute number of events, (3) publication year, (4) log10-transformed event rate, and (5) overall PROBAST risk-of-bias (RoB) score. Each bubble represents an independent validation cohort, and the area of each bubble is proportional to its inverse-variance weight in the random-effects meta-regression. The red dashed line indicates the fitted regression line from the restricted maximum likelihood (REML) model, and the shaded band represents the 95% confidence interval (CI). CI, confidence interval; C-index, concordance index; PROBAST, Prediction model Risk Of Bias Assessment Tool; REML, restricted maximum likelihood; RoB, risk of bias.

Univariable meta-regression (**Figure 4**) showed a significant negative association between the C-index and log10-transformed number of PDAC events (P = 0.012, R² = 42.2%). Validation cohorts with fewer than 50 PDAC events showed greater dispersion in C-index estimates and frequently reported values greater than 0.85. In contrast, estimates from validation cohorts with more than 300 events tended to converge around 0.75-0.80, suggesting potential optimism bias in smaller or event-sparse cohorts. Event-rate patterns were also compatible with spectrum bias: validation cohorts from low-prevalence, population-based settings may better reflect representative performance, whereas estimates from artificially enriched case-control cohorts may overestimate discrimination.

**Figure 5:**
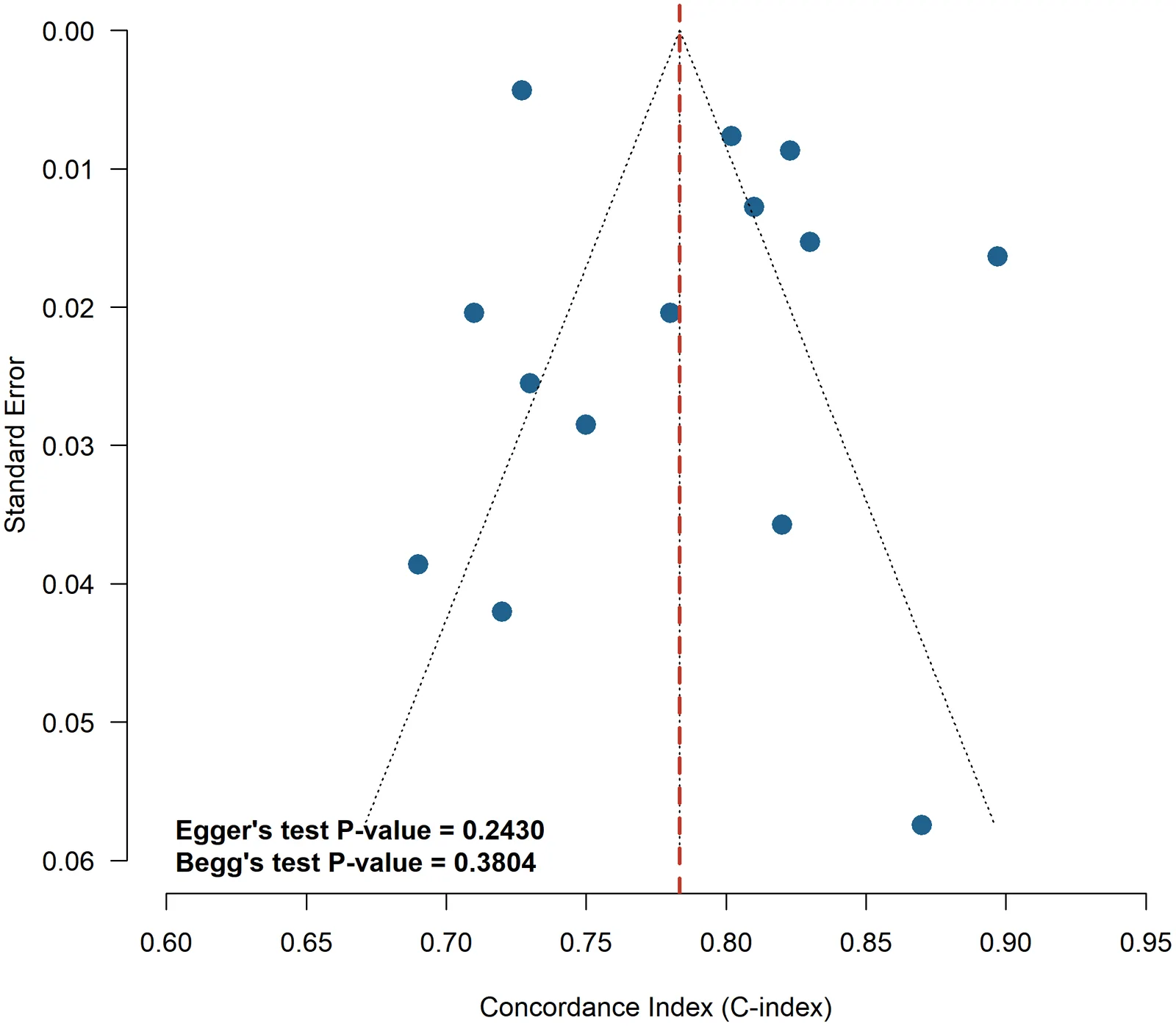
Funnel plot for assessing small-study effects. The plot shows the relationship between reported model discrimination (C-index) and the standard error of the estimate. Each point represents an independent validation cohort. The red dashed vertical line indicates the pooled C-index estimate, and the dotted diagonal lines represent the pseudo-95% confidence limits. Egger’s and Begg’s test P values are displayed in the plot. Because only 14 validation cohort estimates were included and substantial heterogeneity was present, tests for funnel plot asymmetry should be interpreted cautiously. C-index, concordance index; SE, standard error.

Visual inspection of the funnel plot did not suggest strong evidence of small-study effects, and Egger’s and Begg’s test P values are displayed in the plot (**Figure 5**). However, because the analysis included only 14 validation cohort estimates and substantial heterogeneity was present, these tests should be interpreted cautiously.

#### 3.5.4 Sensitivity analyses

**Figure 6:**
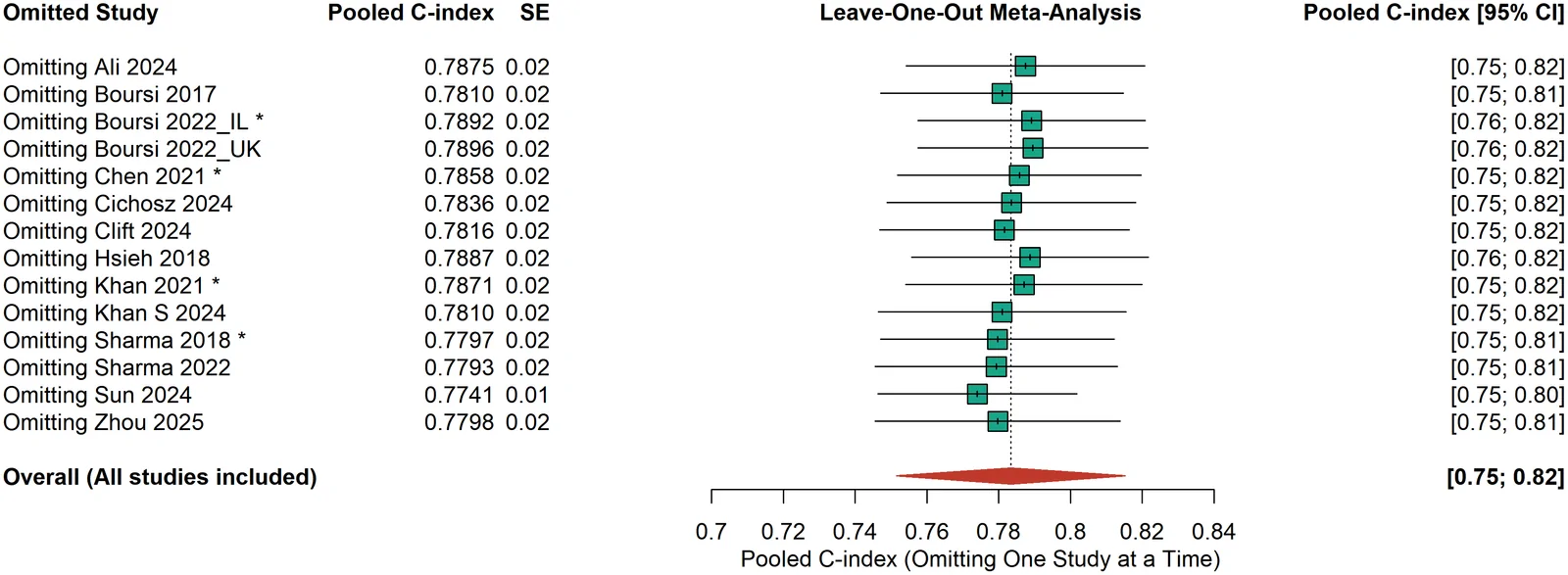
Leave-one-out sensitivity analysis for the pooled overall C-index. Each point represents the pooled C-index obtained after sequential omission of one independent validation cohort, illustrating the leave-one-out sensitivity results for the overall meta-analytic estimate. The dashed reference line indicates the pooled C-index from the full dataset. C-index, concordance index. Green squares and horizontal lines represent leave-one-out pooled estimates and 95% CIs; the red diamond represents the pooled estimate from the full dataset; asterisks indicate cohorts with imputed SEs.

To assess the robustness of the pooled discrimination estimate, we first performed a leave-one-out sensitivity analysis. Sequential omission of individual validation cohorts did not materially change the pooled C-index, suggesting that the overall estimate was not driven by any single validation cohort (**Figure 6**). We further assessed the influence of variance imputation by excluding the four validation cohorts that required standard error estimation using the Hanley-McNeil method: Boursi 2022_IL, Chen 2021, Khan 2021, and Sharma 2018. After these validation cohorts were excluded, the pooled C-index across the remaining 10 validation cohorts with explicitly reported confidence intervals or variance measures was 0.79 (95% CI: 0.76-0.83). This estimate was broadly consistent with the primary pooled C-index of 0.78 (95% CI: 0.75-0.82), suggesting that variance imputation was unlikely to explain the primary pooled estimate. The corresponding forest plot is provided in **Supplementary Figure 9**.

#### 3.5.5 Calibration and clinical utility reporting

Calibration and clinical utility were reported inconsistently across the included studies. Calibration plots were reported in four studies; calibration slope and calibration-in-the-large were reported in two studies each; Hosmer-Lemeshow testing was reported in two studies; and decision-curve analysis was reported in three studies. No study reported an observed/expected ratio. Although many studies reported risk-score cutoffs, risk strata, or proposed risk thresholds, explicit clinical action thresholds linked to downstream imaging, specialist referral, or endoscopic evaluation were rarely defined. Therefore, although the included models showed moderate discrimination, the accuracy of absolute risk prediction and the net clinical benefit of model-guided screening remain uncertain (**Supplementary Table 3**).

### 3.6 Pooled effect sizes of core predictors and qualitative synthesis

**Figure 7:**
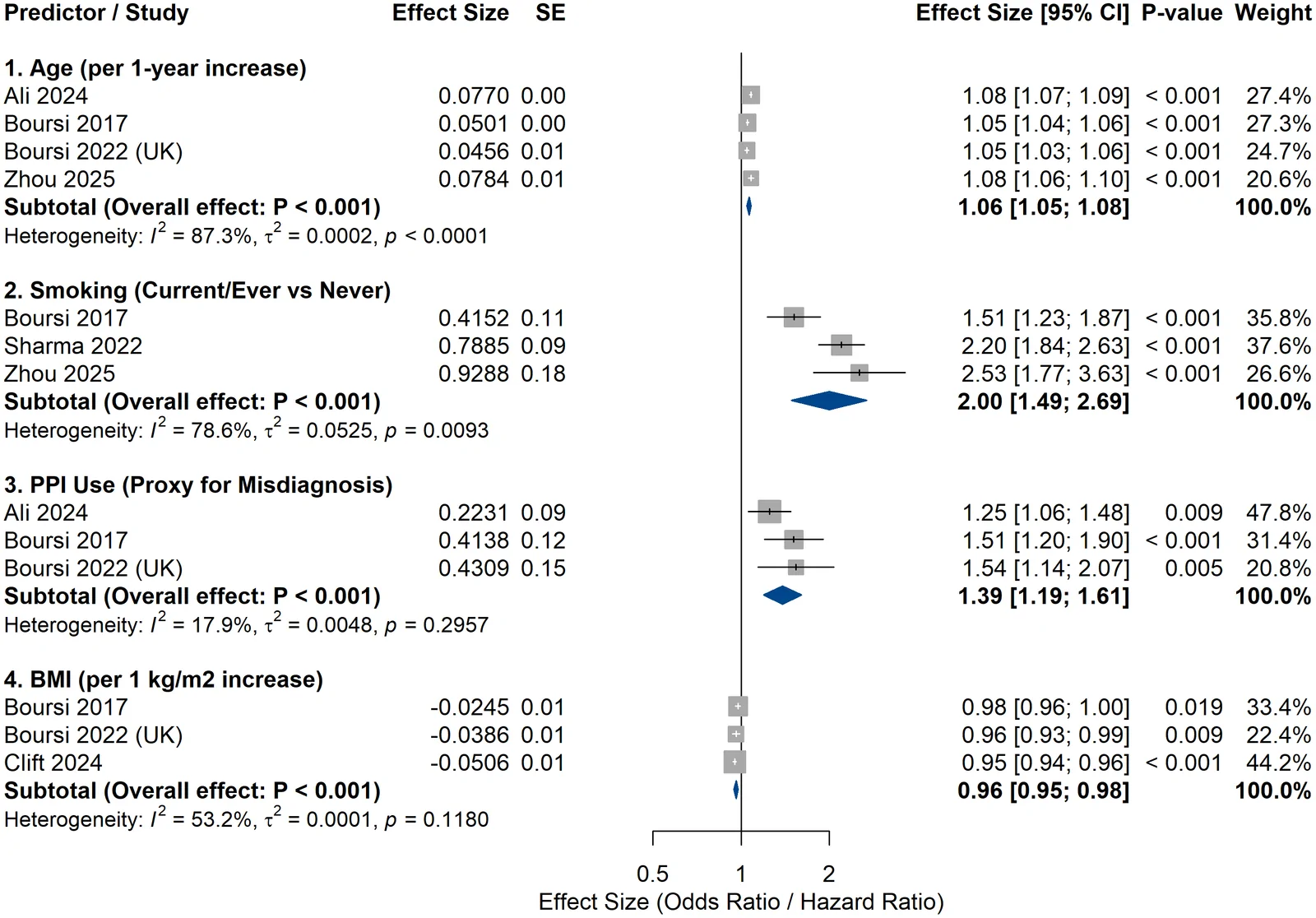
Forest plot of pooled effect sizes for individual predictive features associated with pancreatic cancer risk in patients with NOD. The plot shows pooled exponentiated effect sizes, reported as odds ratios (ORs) or hazard ratios (HRs), for four common predictors: age (per 1-year increase), smoking status (current/ever vs. never), proton pump inhibitor (PPI) use, and body mass index (BMI, per 1 kg/m² increase). Gray squares represent the study-specific effect size estimates, and the horizontal lines through each square indicate the corresponding 95% confidence intervals (CIs). Blue diamonds represent the pooled effect size for each predictor subgroup using a random-effects model. Effect sizes greater than 1 indicate increased risk, whereas effect sizes less than 1 indicate decreased risk. Because odds ratios and hazard ratios are not identical effect measures, these pooled predictor-level estimates should be interpreted as exploratory summaries of direction and relative magnitude rather than as directly interchangeable causal effects. BMI, body mass index; CI, confidence interval; HR, hazard ratio; NOD, new-onset diabetes; OR, odds ratio; PDAC, pancreatic ductal adenocarcinoma; PPI, proton pump inhibitor.

**Figure 8:**
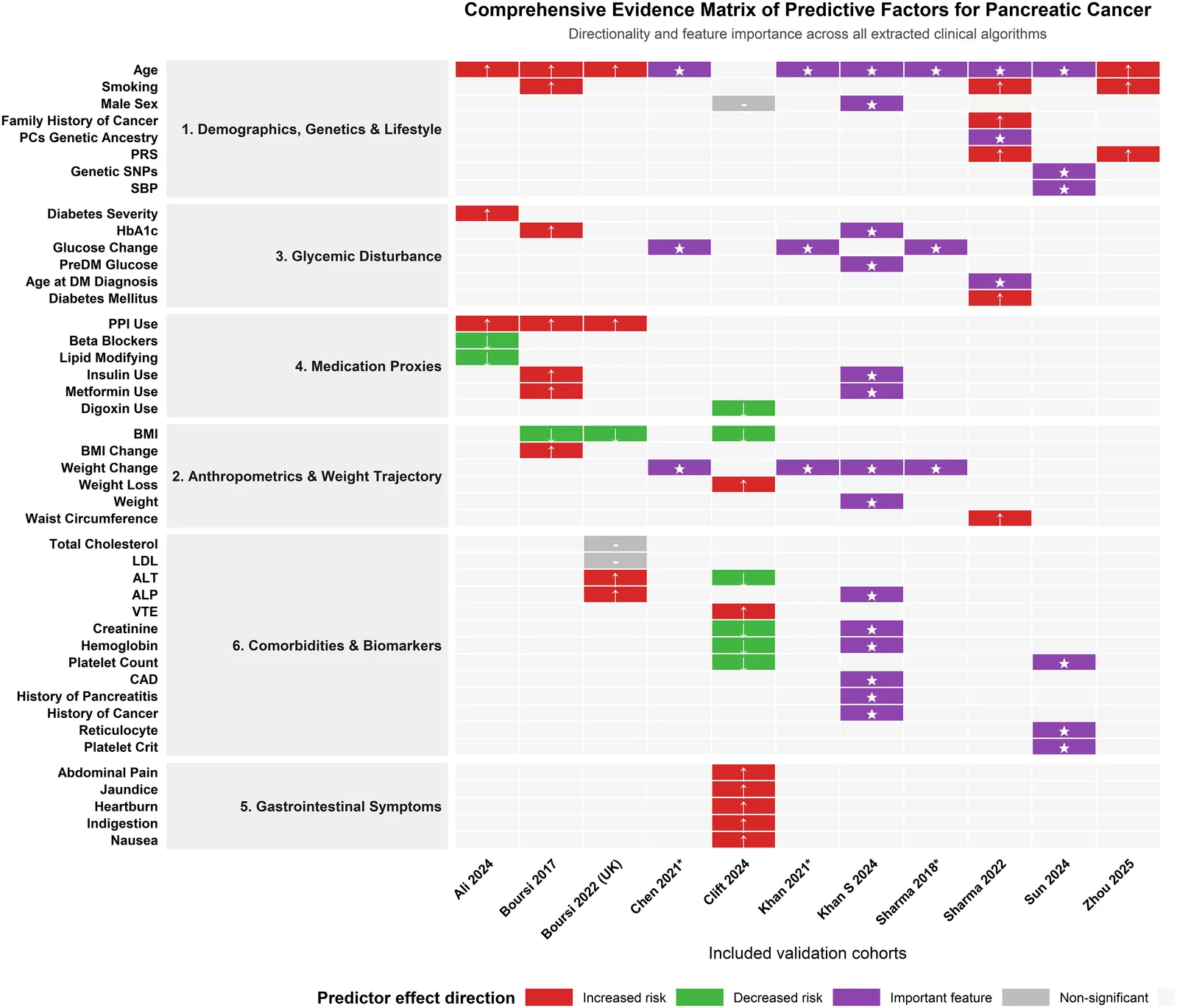
Comprehensive evidence matrix of predictive factors across the included validation cohorts and clinical prediction models. The heatmap summarizes the selection, directionality, and relative importance of predictor variables across included validation cohorts. Color coding indicates the direction or interpretation of each predictor based on the original model reports: red cells indicate predictors associated with increased PDAC risk; green cells indicate predictors associated with decreased PDAC risk; purple cells with asterisks denote features identified as important in machine-learning algorithms; grey cells with dashes indicate variables that were evaluated but were not statistically significant; and blank cells indicate variables that were not evaluated in the corresponding model. Asterisks in validation cohort labels indicate validation cohorts for which the original publications did not report variance data and standard errors were estimated using the Hanley-McNeil method for the discrimination meta-analysis. ALP, alkaline phosphatase; ALT, alanine aminotransferase; BMI, body mass index; CAD, coronary artery disease; DM, diabetes mellitus; HbA1c, glycated hemoglobin; LDL, low-density lipoprotein; ML, machine learning; NOD, new-onset diabetes; PCs, principal components; PDAC, pancreatic ductal adenocarcinoma; PPI, proton pump inhibitor; PRS, polygenic risk score; SBP, systolic blood pressure; SE, standard error; SNPs, single-nucleotide polymorphisms; VTE, venous thromboembolism.

Four core predictors were quantitatively synthesized (**Figure 7**): age (OR = 1.06, 95% CI: 1.05-1.08), smoking history (OR = 2.00, 95% CI: 1.49-2.69), proton pump inhibitor (PPI) use (OR = 1.39, 95% CI: 1.19-1.61), and BMI. Notably, each 1 kg/m² increase in BMI was associated with a 4% lower PDAC risk (OR = 0.96, 95% CI: 0.95-0.98), consistent with the “BMI paradox” described in this setting. Because ORs and HRs are not identical effect measures, these pooled predictor-level estimates should be interpreted as exploratory summaries of direction and relative magnitude rather than as directly interchangeable causal effects. Qualitative heatmap analysis (**Figure 8**) further showed that, across validation cohorts and modeling approaches, rapid weight loss, BMI decline, steep glycemic deterioration, PPI use, smoking, and older age emerged as recurrent predictor signals or important model features. Polygenic risk scores (PRS) may be useful for improving risk stratification beyond conventional clinical predictors.

## 4 Discussion

### 4.1 Main findings and implications for screening

This systematic review and meta-analysis evaluated PDAC risk prediction models in patients with NOD. The pooled C-index was 0.78 (95% CI: 0.75-0.82), suggesting moderate discrimination across the available validation cohorts. However, because substantial heterogeneity was observed across validation cohorts (I² = 95.0%), the pooled estimate should be interpreted as a descriptive summary of published discrimination metrics rather than as a performance estimate that is directly applicable to a specific screening pathway. This heterogeneity likely reflects differences in NOD definitions, age thresholds, prediction windows, model algorithms, predictor selection, data sources, case mix, and validation strategies.

Importantly, this review evaluates temporal prediction rather than causal inference. Because the included models estimate the probability of PDAC diagnosis within a short period after NOD, most commonly within 2–3 years, the identified predictors should be interpreted as markers of occult or preclinical PDAC-associated metabolic disturbance rather than as evidence that NOD causes PDAC onset. The higher discrimination observed in models with 2-year prediction windows compared with models using 3-year or other/unspecified windows may reflect the increasing proximity of NOD-related metabolic changes to an already developing but clinically occult cancer. This interpretation is consistent with reverse causality and should be distinguished from etiologic claims regarding diabetes-induced pancreatic carcinogenesis. Short-term risk prediction may help define a clinically relevant window for targeted surveillance, but prospective external validation, calibration assessment, and clinical utility evaluation are required before these models are used in screening pathways.

### 4.2 Methodological considerations: variance imputation and optimism bias

The Hanley-McNeil method allowed inclusion of validation cohorts that did not report variance measures for the C-index or AUC. However, this approach relies on assumptions of independent observations and an approximately normal distribution of the AUC estimate. We therefore performed a sensitivity analysis excluding validation cohorts with imputed standard errors. The pooled C-index after exclusion of these validation cohorts was 0.79 (95% CI: 0.76-0.83), which was broadly consistent with the primary pooled estimate of 0.78 (95% CI: 0.75-0.82). This finding suggests that the overall discrimination estimate was not primarily driven by variance imputation, although the pooled result should still be interpreted cautiously because of substantial heterogeneity.

Our meta-regression also suggested potential optimism bias in small or event-sparse validation cohorts. Validation cohorts with fewer PDAC events showed greater dispersion and sometimes reported higher C-indices, whereas estimates from larger validation cohorts tended to converge around 0.75-0.80. This pattern is consistent with overfitting and low events-per-variable ratios in prediction modeling, as emphasized by TRIPOD and PROBAST guidance (29). These findings support the need for larger, adequately powered validation cohorts and transparent reporting of model development, validation, and calibration.

### 4.3 Possible biological interpretation of core predictors

The high-frequency clinical predictors identified in this review should be interpreted as potential markers of tumor-associated host metabolic remodeling rather than as causal determinants of PDAC onset. Unintentional weight loss, BMI decline, and rapid glycemic deterioration may reflect catabolic and endocrine changes in the setting of occult or preclinical PDAC (31). Recent metabolic kinetic studies indicate that PDAC-related diabetes differs from ordinary type 2 diabetes in several aspects of glucose homeostasis, including β-cell dysfunction and hyperinsulinemia (32). In experimental settings, hyperinsulinemia and insulin receptor signaling in pancreatic acinar cells have been linked to local inflammation and early pancreatic neoplastic changes (33, 34). However, the present meta-analysis cannot determine whether these pathways cause PDAC onset in patients with NOD. Rather, these data provide biological plausibility for why clinical variables such as glycemic deterioration, weight loss, BMI decline, and PPI use may help identify patients with NOD who already harbor occult or preclinical PDAC. PPI use may also reflect nonspecific gastrointestinal symptoms caused by early tumor-related effects, although it should be interpreted as a clinical proxy rather than a causal factor (35).

### 4.4 Hypothesis-generating metabolic and oncogenic pathways related to occult PDAC in NOD

mTOR signaling may provide a hypothesis-generating biological framework for interpreting why some patients with NOD show clinical changes before PDAC is diagnosed. This framework should not be interpreted as evidence that NOD causes PDAC onset. As a central nutrient- and growth factor-sensing pathway, mTOR integrates amino acid availability, glucose metabolism, insulin/IGF signaling, mitochondrial function, protein synthesis, autophagy, and cellular growth through mTORC1 and mTORC2. In patients with NOD, rapid changes in glycemic control, insulin secretion, insulin resistance, amino acid metabolism, and nutritional status may reflect systemic metabolic disturbances associated with occult or preclinical PDAC.

Recent studies support the relevance of amino acid–mTORC1–PDX1 signaling to pancreatic β-cell function, showing that mTORC1-mediated phosphorylation of PDX1 enhances insulin expression and β-cell proliferation, while amino acids may regulate systemic glucose homeostasis through the Rab1A–mTORC1–p-PDX1 axis (36, 37). These findings provide biological plausibility for linking β-cell dysfunction and glycemic deterioration with metabolic dysregulation, but they do not establish causality between NOD and PDAC onset in the present review. In PDAC-related metabolic remodeling, insulin resistance and hyperinsulinemia may be associated with insulin/IGF–PI3K–AKT–mTOR signaling, inflammatory responses, and tumor adaptation. Nuclear mTOR has also been implicated in transcriptional, epigenetic, and chromatin-remodeling programs related to cellular growth and metabolism (38).

**Figure 9:**
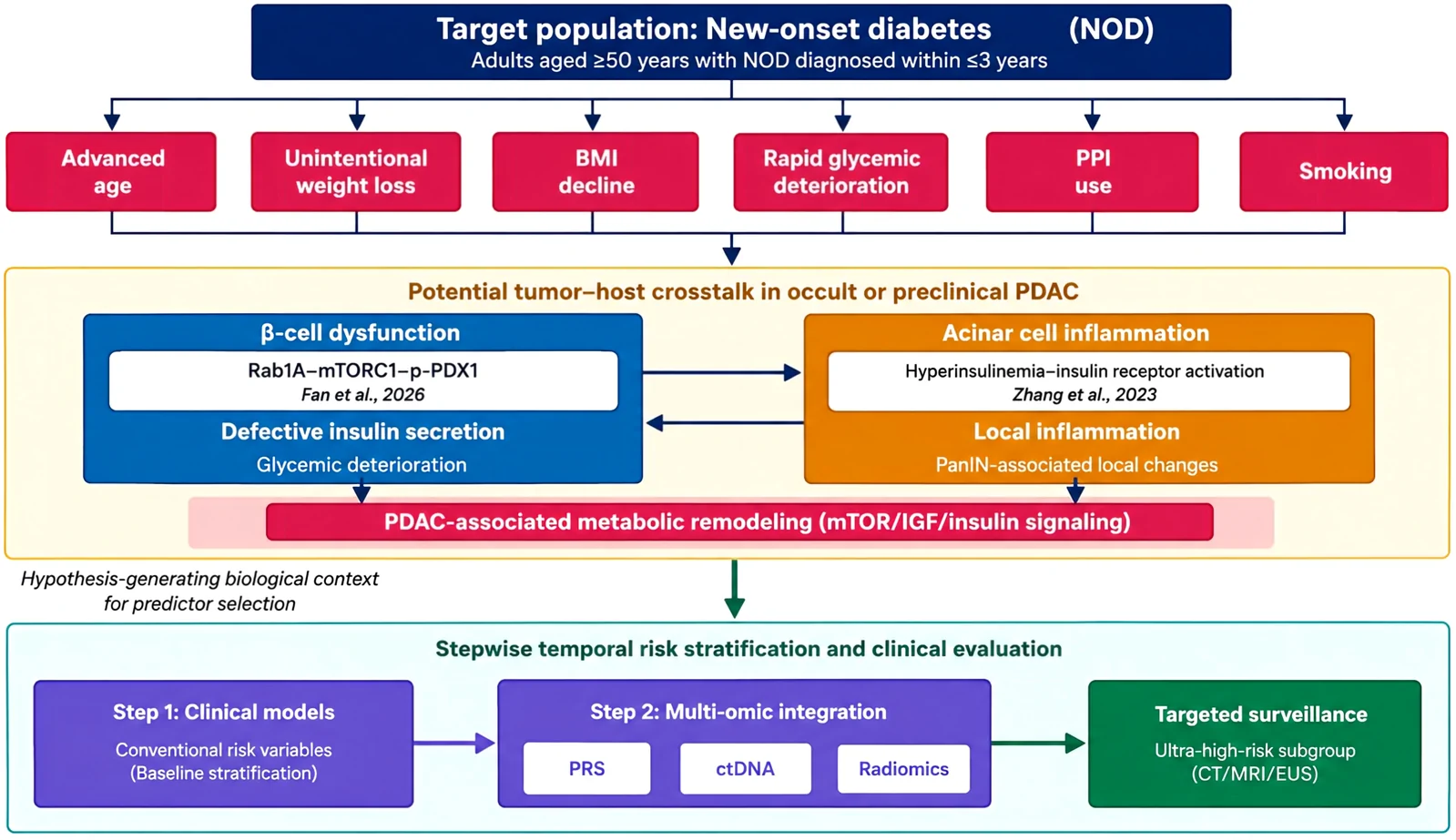
Conceptual framework for temporal risk stratification of occult or preclinical pancreatic ductal adenocarcinoma (PDAC) in patients with new-onset diabetes (NOD). The schematic illustrates a hypothesis-generating framework linking clinical recognition, possible biological context, and potential targeted imaging surveillance. The target population comprises adults aged ≥50 years with NOD diagnosed within ≤3 years. High-risk clinical warning signals include advanced age, unintentional weight loss, BMI decline, rapid glycemic deterioration, PPI use, and smoking. These clinical features should be interpreted as potential markers of occult or preclinical PDAC-associated metabolic disturbance rather than as evidence that NOD causes PDAC onset. The middle panel summarizes possible tumor-host metabolic crosstalk, including β-cell dysfunction, the Rab1A-mTORC1-p-PDX1 axis, hyperinsulinemia, insulin receptor activation, local inflammation, and mTOR/IGF/insulin signaling. These pathways provide biological plausibility for predictor selection but do not establish causal relationships in the present review. Grounded in this hypothesis-generating framework, Step 1 uses conventional clinical-variable models to establish baseline risk stratification, and Step 2 may integrate multi-omic dimensions, including polygenic risk scores, circulating tumor DNA, and AI-assisted radiomics, to further refine risk stratification after validation. A potential clinical objective is to identify an ultra-high-risk subgroup that could be considered for targeted imaging surveillance, such as CT, MRI, or EUS, after prospective validation and clinical utility assessment. Arrows indicate proposed associations and workflow links rather than causal pathways. AI, artificial intelligence; BMI, body mass index; CT, computed tomography; ctDNA, circulating tumor DNA; EUS, endoscopic ultrasound; IGF, insulin-like growth factor; mTOR, mechanistic target of rapamycin; mTORC1, mechanistic target of rapamycin complex 1; NOD, new-onset diabetes; PanIN, pancreatic intraepithelial neoplasia; PDAC, pancreatic ductal adenocarcinoma; PDX1, pancreatic and duodenal homeobox 1; PPI, proton pump inhibitor; PRS, polygenic risk score.

The present review did not evaluate mTOR biomarkers directly. These pathways should therefore be viewed as hypothesis-generating biological context for clinical risk stratification rather than as mechanistic proof of cancer initiation. Pancreatic tumorigenesis is unlikely to be explained by a single pathway; inflammatory mediators, insulin/IGF signaling, KRAS-driven metabolism, Hedgehog-related developmental signaling, and mTOR-related nutrient sensing may interact during preclinical tumor evolution. Although the Hedgehog/Ptc/PKAc study does not directly address mTOR signaling, it supports the broader concept of developmental and oncogenic signaling crosstalk (39). Accordingly, **Figure 9** presents a conceptual framework for temporal risk stratification of occult or preclinical PDAC in NOD rather than a causal pathway from NOD to PDAC onset.

### 4.5 Future directions: integration of multi-omics and dynamic risk assessment

Our analyses suggest that discrimination based solely on conventional electronic health-record variables may have limited room for improvement within the current evidence base. Future progress may require integration of biomarker, imaging, and multi-omic data. Recent Mendelian randomization studies have reported genetic associations between specific T2DM-related clusters, such as lipodystrophy-related mechanisms, and PDAC risk (40). Future studies should evaluate whether dynamic clinical trajectories, circulating tumor DNA (ctDNA) (41), polygenic risk scores (PRS) (26, 28), and AI-assisted CT radiomics (42) can improve risk stratification. Model development should follow TRIPOD guidance. High discrimination alone does not ensure accurate absolute risk prediction in real-world practice; therefore, calibration should be routinely evaluated and reported alongside discrimination metrics (29, 30). Without calibration and decision-curve evidence, the clinical consequences of applying a given risk threshold for CT, MRI, endoscopic ultrasound, or specialist referral remain uncertain.

Although Random Forest and XGBoost models showed numerically higher discrimination in the included studies, their clinical translation remains uncertain because interpretability, calibration reporting, external validation, and implementation details were limited. In the included machine-learning studies, reporting of SHAP values, permutation-based feature importance, calibration across risk strata, independent external validation, and reproducible deployment procedures was incomplete. Future machine-learning studies should report both global and patient-level interpretability analyses, including SHAP values, permutation-based feature importance, and partial dependence plots, to clarify which predictors drive individual risk estimates (43). Reports of model deployment should also specify data availability, missing-data handling, update frequency, referral thresholds, explanations that clinicians can review when interpreting individual risk estimates, workflow integration, and prospective monitoring for calibration drift (44). Without these elements, high C-index values alone are insufficient to support routine use in NOD-based PDAC screening pathways. Before clinical deployment, machine-learning models require independent external validation and calibration assessment (30), and their clinical utility should be evaluated using decision-curve analysis (45).

### 4.6 Strengths and limitations

This review has several strengths, including the use of meta-regression to explore potential optimism bias and sensitivity analysis to examine the influence of missing variance data. Several limitations should also be considered. Most included studies relied on internal validation only, which may overestimate model performance and does not establish transportability across healthcare systems, coding practices, or patient populations. Although we assessed potential overlap according to data source, study period, geography, and cohort characteristics, overlap could not be fully excluded for some studies using large routine-care databases or biobank-derived populations. PROBAST evaluation indicated that calibration was frequently underreported in the original studies, despite its importance for real-world risk estimation (11, 30). Evidence from independent external validation was limited and concentrated mainly in ENDPAC-related cohorts (17, 19, 23, 25), whereas Clift 2024 provided internal-external validation for QPancreasD (21).

## 5 Conclusion

Existing PDAC risk prediction models for patients with NOD showed moderate discriminative performance in published validation cohorts, but this estimate should not be interpreted as directly generalizable to routine screening settings because of substantial clinical and methodological heterogeneity. The core early-warning profile consists of older age, smoking history, unintentional weight loss or BMI decline, steep glycemic deterioration, and nonspecific gastrointestinal symptoms reflected by PPI use. Current models based mainly on conventional clinical data may provide limited additional discrimination within the available evidence base. Furthermore, potential optimism bias in small cohorts or cohorts evaluated by internal validation only, together with insufficient evidence on calibration, clinical utility, and independent external validation, limits immediate clinical implementation. Mechanistically, the association between NOD-related clinical warning signs and subsequent PDAC diagnosis may reflect tumor-related metabolic remodeling, β-cell dysfunction, systemic inflammation, hyperinsulinemia, and mTOR/IGF/insulin-related signaling pathways; however, these findings should be interpreted as hypothesis-generating biological context rather than as evidence that NOD causes PDAC onset. Future studies should follow TRIPOD guidance, use large multicenter cohorts, report calibration and decision-curve analyses, perform independent external validation and recalibration, and evaluate whether dynamic clinical trajectories combined with multi-omic biomarkers such as PRS, ctDNA, radiomics, and potentially mTOR-related metabolic signatures can improve temporal risk stratification.

## Data Availability

The original contributions presented in the study are included in the article and **Supplementary Material**. Further inquiries can be directed to the corresponding authors.

## References

[1] Siegel RL Giaquinto AN Jemal A . Cancer statistics, 2024. CA Cancer J Clin. (2024) 74:12–49. doi: 10.3322/caac.21820 38230766

[2] Mannucci A Goel A . Advances in pancreatic cancer early diagnosis, prevention, and treatment: the past, the present, and the future. CA Cancer J Clin. (2026) 76:e70035. doi: 10.3322/caac.70035 40971231 PMC12788368

[3] US Preventive Services Task Force . Screening for pancreatic cancer: US Preventive Services Task Force reaffirmation recommendation statement. JAMA. (2019) 322:438–44. doi: 10.1001/jama.2019.10232 31386141

[4] Canto MI Harinck F Hruban RH Offerhaus GJ Poley JW Kamel I . International Cancer of the Pancreas Screening (CAPS) Consortium summit on the management of patients with increased risk for familial pancreatic cancer. Gut. (2013) 62:339–47. doi: 10.1136/gutjnl-2012-303108 23135763 PMC3585492

[5] Chari ST Wu B Lopez C Lustigova E Chen Q Van Den Eeden SK . Risk of pancreatic cancer in glycemically defined new-onset diabetes: a prospective cohort study. Gastroenterology. (2026) 170:106–17. doi: 10.1053/j.gastro.2025.06.025 40633624 PMC12403438

[6] Roy A Sahoo J Kamalanathan S Naik D Mohan P Kalayarasan R . Diabetes and pancreatic cancer: exploring the two-way traffic. World J Gastroenterol. (2021) 27:4939–62. doi: 10.3748/wjg.v27.i30.4939 34497428 PMC8384733

[7] Hart PA Andersen DK Petrov MS Goodarzi MO . Distinguishing diabetes secondary to pancreatic diseases from type 2 diabetes mellitus. Curr Opin Gastroenterol. (2021) 37:520–5. doi: 10.1097/MOG.0000000000000754 34265796 PMC8364493

[8] Gallo M Adinolfi V Morviducci L Acquati S Tuveri E Ferrari P . Early prediction of pancreatic cancer from new-onset diabetes: an AIOM/AMD/SIE/SIF multidisciplinary consensus position paper. ESMO Open. (2021) 6:100155. doi: 10.1016/j.esmoop.2021.100155 34020401 PMC8144346

[9] Schwartz NRM Matrisian LM Shrader EE Feng Z Chari S Roth JA . Potential cost-effectiveness of risk-based pancreatic cancer screening in patients with new-onset diabetes. J Natl Compr Canc Netw. (2022) 20:451–9. doi: 10.6004/jnccn.2020.7798 34153945

[10] Santos R Coleman HG Cairnduff V Kunzmann AT . Clinical prediction models for pancreatic cancer in general and at-risk populations: a systematic review. Am J Gastroenterol. (2023) 118:26–40. doi: 10.14309/ajg.0000000000002022 36148840

[11] Moons KGM Wolff RF Riley RD Whiting PF Westwood M Collins GS . PROBAST: a tool to assess risk of bias and applicability of prediction model studies. Ann Intern Med. (2019) 170:W1–W33. doi: 10.7326/M18-1377 30596876

[12] Cong H Song J Liu L Liu S Wu H Nan Z . Risk factors and early prediction of pancreatic cancer among patients with diabetes mellitus: a systematic review and meta-analysis. Front Endocrinol (Lausanne). (2025) 16:1698850. doi: 10.3389/fendo.2025.1698850 41347135 PMC12673663

[13] Hajibandeh S Intrator C Carrington-Windo E James R Hughes I Hajibandeh S . Accuracy of the END-PAC model in predicting the risk of developing pancreatic cancer in patients with new-onset diabetes: a systematic review and meta-analysis. Biomedicines. (2023) 11:3040. doi: 10.3390/biomedicines11113040 38002040 PMC10669673

[14] Hanley JA McNeil BJ . The meaning and use of the area under a receiver operating characteristic (ROC) curve. Radiology. (1982) 143:29–36. doi: 10.1148/radiology.143.1.7063747 7063747

[15] Ali S Coory M Donovan P Na R Pandeya N Pearson SA . Predicting the risk of pancreatic cancer in women with new-onset diabetes mellitus. J Gastroenterol Hepatol. (2024) 39:1057–64. doi: 10.1111/jgh.16503 38373821

[16] Boursi B Finkelman B Giantonio BJ Haynes K Rustgi AK Rhim AD . A clinical prediction model to assess risk for pancreatic cancer among patients with new-onset diabetes. Gastroenterology. (2017) 152:840–850.e3. doi: 10.1053/j.gastro.2016.11.046 27923728 PMC5337138

[17] Boursi B Patalon T Webb M Margalit O Beller T Yang YX . Validation of the enriching new-onset diabetes for pancreatic cancer model: a retrospective cohort study using real-world data. Pancreas. (2022) 51:196–9. doi: 10.1097/MPA.0000000000002000 35404897

[18] Boursi B Finkelman B Giantonio BJ Haynes K Rustgi AK Rhim AD . A clinical prediction model to assess risk for pancreatic cancer among patients with prediabetes. Eur J Gastroenterol Hepatol. (2022) 34:33–8. doi: 10.1097/MEG.0000000000002052 33470698 PMC8286263

[19] Chen W Butler RK Lustigova E Chari ST Wu BU . Validation of the enriching new-onset diabetes for pancreatic cancer model in a diverse and integrated healthcare setting. Dig Dis Sci. (2021) 66:78–87. doi: 10.1007/s10620-020-06139-z 32112260

[20] Cichosz SL Jensen MH Hejlesen O Henriksen SD Drewes AM Olesen SS . Prediction of pancreatic cancer risk in patients with new-onset diabetes using a machine learning approach based on routine biochemical parameters. Comput Methods Programs BioMed. (2024) 244:107965. doi: 10.1016/j.cmpb.2023.107965 38070389

[21] Clift AK Tan PS Patone M Liao W Coupland C Bashford-Rogers R . Predicting the risk of pancreatic cancer in adults with new-onset diabetes: development and internal-external validation of a clinical risk prediction model. Br J Cancer. (2024) 130:1969–78. doi: 10.1038/s41416-024-02693-9 38702436 PMC11183048

[22] Hsieh MH Sun LM Lin CL Hsieh MJ Hsu CY Kao CH . Development of a prediction model for pancreatic cancer in patients with type 2 diabetes using logistic regression and artificial neural network models. Cancer Manag Res. (2018) 10:6317–24. doi: 10.2147/CMAR.S180791 30568493 PMC6267763

[23] Khan S Safarudin RF Kupec JT . Validation of the ENDPAC model: identifying new-onset diabetics at risk of pancreatic cancer. Pancreatology. (2021) 21:550–5. doi: 10.1016/j.pan.2021.02.001 33583686 PMC8393564

[24] Khan S Bhushan B . Machine learning predicts patients with new-onset diabetes at risk of pancreatic cancer. J Clin Gastroenterol. (2024) 58:681–91. doi: 10.1097/MCG.0000000000001897 37522752

[25] Sharma A Kandlakunta H Nagpal SJS Feng Z Hoos W Petersen GM . Model to determine risk of pancreatic cancer in patients with new-onset diabetes. Gastroenterology. (2018) 155:730–739.e3. doi: 10.1053/j.gastro.2018.05.023 29775599 PMC6120785

[26] Sharma S Tapper WJ Collins A Hamady ZZR . Predicting pancreatic cancer in the UK Biobank cohort using polygenic risk scores and diabetes mellitus. Gastroenterology. (2022) 162:1665–1674.e2. doi: 10.1053/j.gastro.2022.01.016 35065983

[27] Sun Y Hu C Hu S Xu H Gong J Wu Y . Predicting pancreatic cancer in new-onset diabetes cohort using a novel model with integrated clinical and genetic indicators: a large-scale prospective cohort study. Cancer Med. (2024) 13:e70388. doi: 10.1002/cam4.70388 39526476 PMC11551786

[28] Zhou Y Wu Z Zeng L Chen R . Combining genetic and non-genetic factors to predict the risk of pancreatic cancer in patients with new-onset diabetes mellitus. BMC Med. (2025) 23:224. doi: 10.1186/s12916-025-04048-4 40234846 PMC12001390

[29] Collins GS Reitsma JB Altman DG Moons KG . Transparent reporting of a multivariable prediction model for individual prognosis or diagnosis (TRIPOD): the TRIPOD statement. BMJ. (2015) 350:g7594. doi: 10.1136/bmj.g7594 25569120

[30] Van Calster B McLernon DJ van Smeden M Wynants L Steyerberg EW Topic Group “Evaluating diagnostic tests and prediction models” of the STRATOS initiative . Calibration: the Achilles heel of predictive analytics. BMC Med. (2019) 17:230. doi: 10.1186/s12916-019-1466-7 31842878 PMC6912996

[31] Binang HB Perera CJ Apte MV . Role of pancreatic tumour-derived exosomes and their cargo in pancreatic cancer-related diabetes. Int J Mol Sci. (2023) 24:10203. doi: 10.3390/ijms241210203 37373351 PMC10299712

[32] Toledo FGS Li Y Wang F Bellin MD Brand R Cusi K . Pancreatic cancer-related diabetes and type 2 diabetes differ in multiple aspects of glucose homeostasis. Diabetologia. (2025) 68:2854–66. doi: 10.1007/s00125-025-06543-y 40991017 PMC12579526

[33] Zhang AMY Xia YH Lin JSH Chu KH Wang WCK Ruiter TJJ . Hyperinsulinemia acts via acinar insulin receptors to initiate pancreatic cancer by increasing digestive enzyme production and inflammation. Cell Metab. (2023) 35:2119–2135.e5. doi: 10.1016/j.cmet.2023.10.003 37913768

[34] Post Z Rosario Lora D Blogowski W . Unraveling the sweet connection between pancreatic cancer and hyperglycemia. Trends Endocrinol Metab. (2026) 37:230–44. doi: 10.1016/j.tem.2025.06.003 40603165

[35] Ruze R Song J Yin X Chen Y Xu R Wang C . Mechanisms of obesity- and diabetes mellitus-related pancreatic carcinogenesis: a comprehensive and systematic review. Signal Transduct Target Ther. (2023) 8:139. doi: 10.1038/s41392-023-01376-w 36964133 PMC10039087

[36] Fan J Zhang X Zhang J Zhao T Burley SK Zheng XFS . PDX1 phosphorylation at S61 by mTORC1 links nutrient signaling to β cell function and metabolic disease. Cell Rep. (2026) 45:116811. doi: 10.1016/j.celrep.2025.116811 41528843 PMC12949489

[37] Fan J Yuan Z Burley SK Libutti SK Zheng XFS . Amino acids control blood glucose levels through mTOR signaling. Eur J Cell Biol. (2022) 101:151240. doi: 10.1016/j.ejcb.2022.151240 35623230 PMC10035058

[38] Zhao T Fan J Abu-Zaid A Burley SK Zheng XFS . Nuclear mTOR signaling orchestrates transcriptional programs underlying cellular growth and metabolism. Cells. (2024) 13:781. doi: 10.3390/cells13090781 38727317 PMC11083943

[39] Fan J Gao Y Lu Y Wu W Yuan S Wu H . PKAc-directed interaction and phosphorylation of Ptc is required for Hh signaling inhibition in Drosophila. Cell Discov. (2019) 5:44. doi: 10.1038/s41421-019-0112-z 31636957 PMC6796939

[40] Zhang T Hua X Mohindroo C Wang X Dutta D Liu J . Different diabetes types and pancreatic ductal adenocarcinoma: a Mendelian randomization and pathway/gene-set analysis. J Natl Cancer Inst. (2026) 118:437–47. doi: 10.1093/jnci/djaf308 41206949 PMC13005432

[41] Wang X Wang H Zhang M Li H Liu Y Huang H . Development and prospective validation of a cell-free DNA-based model for the early detection of pancreatic cancer. Cancer Discov. (2026) 16:66–80. doi: 10.1158/2159-8290.CD-25-0323 40982573

[42] Zhao G Chen X Zhu M Liu Y Wang Y . Exploring the application and future outlook of artificial intelligence in pancreatic cancer. Front Oncol. (2024) 14:1345810. doi: 10.3389/fonc.2024.1345810 38450187 PMC10915754

[43] Lundberg SM Erion G Chen H DeGrave A Prutkin JM Nair B . From local explanations to global understanding with explainable AI for trees. Nat Mach Intell. (2020) 2:56–67. doi: 10.1038/s42256-019-0138-9 32607472 PMC7326367

[44] Kelly CJ Karthikesalingam A Suleyman M Corrado G King D . Key challenges for delivering clinical impact with artificial intelligence. BMC Med. (2019) 17:195. doi: 10.1186/s12916-019-1426-2 31665002 PMC6821018

[45] Vickers AJ Elkin EB . Decision curve analysis: a novel method for evaluating prediction models. Med Decis Making. (2006) 26:565–74. doi: 10.1177/0272989X06295361 17099194 PMC2577036

