# Supplementary material for: Performance of risk prediction models for pancreatic cancer in patients with new-onset diabetes: a systematic review and meta-analysis
Source: Front Oncol. 2026 Aug 18;16:1868121. doi: 10.3389/fonc.2026.1868121 (PMC13531343; doi:10.3389/fonc.2026.1868121)
Supplement: Supplementary file 2 [file DataSheet2.docx]

Supplementary Material

**Supplementary Figure S1. Forest plot of the pooled C-index stratified by study design.**

The included validation cohorts were stratified into retrospective (k = 11; Refs. [15-25]) and prospective (k = 3; Refs. [26-28]) designs. Pooled estimates were calculated using a random-effects model.

**Supplementary Figure S2. Forest plot of the pooled C-index stratified by prediction window.**

Subgroup analysis comparing models designed to predict pancreatic cancer within a 2-year window (k = 3; Refs. [21, 26, 27]), a 3-year window (k = 9; Refs. [15-20, 24, 25, 28]), and other/unspecified time frames (k = 2; Refs. [22, 23]).

**Supplementary Figure S3. Forest plot of the pooled C-index stratified by target population.**

The cohorts were stratified into general new-onset diabetes (NOD) populations with broad age ranges (k = 7; Refs. [16, 18, 21, 22, 26-28]) versus ultra high-risk populations defined by age ≥ 50 years (k = 7; Refs. [15, 17, 19, 20, 23-25]).

**Supplementary Figure S4. Forest plot of the pooled C-index stratified by validation method.**

Subgroup analysis comparing models evaluated via internal validation only (k = 10; Refs. [15, 16, 18, 20, 22, 24-28]) versus those subjected to independent external or internal-external cross-validation (k = 4; Refs. [17, 19, 21, 23]).

**Supplementary Figure S5. Forest plot of the pooled C-index stratified by model algorithm type.**

The incorporated algorithms were categorized into standard logistic regression (k = 7; Refs. [15, 16, 18, 22, 25-27]), external validation models (k = 3; Refs. [17, 19, 23]), machine learning algorithms (k = 2; Refs. [20, 24]), and time-to-event Cox proportional hazards regression (k = 2; Refs. [21, 28]).

**Supplementary Figure S6. Forest plot of the pooled C-index stratified by geographical region.**

Subgroup analysis grouping validation cohorts based on their geographical origins: Europe (k = 6; Refs. [16, 18, 20, 21, 26, 27]), North America (k = 4; Refs. [19, 23-25]), Asia (k = 3; Refs. [17, 22, 28]), and Oceania (k = 1; Ref. [15]).

**Supplementary Figure S7. Forest plot of the pooled C-index stratified by data source type.**

The analysis compares models derived from single databases or deep-phenotyped cohorts (k = 9; Refs. [16-19, 21, 25-28]), federated clinical networks (k = 2; Refs. [23, 24]), and broad national administrative registries (k = 3; Refs. [15, 20, 22]).

**Supplementary Figure S8. Forest plot of the pooled C-index stratified by overall risk of bias.**

Subgroup analysis comparing prognostic models assessed as having a high risk of bias (k = 10; Refs. [15, 17, 19, 20, 22-27]) versus a low risk of bias (k = 4; Refs. [16, 18, 21, 28]), according to the Prediction model Risk Of Bias Assessment Tool (PROBAST).
