# Supplementary material for: Performance of risk prediction models for pancreatic cancer in patients with new-onset diabetes: a systematic review and meta-analysis
Source: Front Oncol. 2026 Aug 18;16:1868121. doi: 10.3389/fonc.2026.1868121 (PMC13531343; doi:10.3389/fonc.2026.1868121)
Supplement: Supplementary file 3 [file DataSheet3.docx]

Supplementary Material

**Supplementary Table S1.** **Estimation of missing standard errors (SE) and 95% confidence intervals for included studies.**

| **Study ID** | **Total (n)** | **Events (n)** | **C-index** | **Estimated SE** | **Estimated 95% CI** |
| --- | --- | --- | --- | --- | --- |
| **Boursi2022(IL)(17)** | **5408** | **59** | **0.69** | **0.0386** | **0.61–0.77** |
| **Chen 2021(19)** | **13947** | **99** | **0.75** | **0.0285** | **0.69–0.81** |
| **Khan 2021(23)** | **6302** | **48** | **0.72** | **0.0420** | **0.64–0.80** |
| **Sharma 2018(25)** | **1561** | **16** | **0.87** | **0.0574** | **0.76–0.98** |

Notes: For studies that did not report original variance data, the standard error (SE) of the C-index was algebraically estimated using the Hanley-McNeil formula based on the total sample size and the number of events. The estimated 95% confidence intervals (CI) were subsequently derived using the formula: C-index ± (1.96 × Estimated SE). Abbreviations: CI, confidence interval; SE, standard error.

**Supplementary Table S2.Risk of Bias and Applicability Assessment of Included Studies based on the PROBAST tool.**

| **Study ID** | **D1: Participants** | **D2: Predictors** | **D3: Outcome** | **D4: Analysis** | **Overall Risk of Bias** | **A1: Participants** | **A2: Predictors** | **A3: Outcome** | **Overall Applicability Concerns** |
| --- | --- | --- | --- | --- | --- | --- | --- | --- | --- |
| **Ali 2024(15)** | Low | Low | Low | High | **High** | Low | Unclear | Low | **Unclear** |
| **Boursi 2017(16)** | Low | Low | Low | Low | **Low** | Low | Low | Low | **Low** |
| **Boursi 2022 (IL)(17)** | Low | Low | Low | High | **High** | Low | Low | Low | **Low** |
| **Boursi 2022 (UK)(18)** | Low | Low | Low | Low | **Low** | Low | Low | Low | **Low** |
| **Chen 2021(19)** | Low | High | Low | High | **High** | Low | High | Low | **High** |
| **Cichosz 2024(20)** | High | Low | Low | High | **High** | High | Low | Low | **High** |
| **Clift 2024(21)** | Low | Low | Low | Low | **Low** | Low | Low | Low | **Low** |
| **Hsieh 2018(22)** | Low | Low | Low | High | **High** | Low | Unclear | Low | **Unclear** |
| **Khan 2021(23)** | High | High | Low | High | **High** | High | High | Low | **High** |
| **KhanS 2024(24)** | Low | Low | Low | High | **High** | Low | Low | Low | **Low** |
| **Sharma 2018(25)** | Low | Low | Low | High | **High** | Low | Low | Low | **Low** |
| **Sharma 2022(26)** | Low | High | High | High | **High** | Low | High | Low | **High** |
| **Sun 2024(27)** | Low | Low | Unclear | High | **High** | Low | Low | Low | **Low** |
| **Zhou 2025(28)** | Low | Low | Low | Low | **Low** | Low | Low | Low | **Low** |

Abbreviations: D1–D4, Domains 1 to 4 of the PROBAST tool for risk of bias; A1–A3, Applicability Domains 1 to 3; PROBAST, Prediction model Risk Of Bias ASsessment Tool.

Notes: A "High" overall risk of bias was frequently assigned in the Analysis domain (D4). This was largely driven by retrospective study designs with inadequate handling of missing data, a lack of internal/external validation, or the absence of reported variance (confidence intervals) for performance metrics in the original publications.
