# Supplementary material for: Performance of risk prediction models for pancreatic cancer in patients with new-onset diabetes: a systematic review and meta-analysis
Source: Front Oncol. 2026 Aug 18;16:1868121. doi: 10.3389/fonc.2026.1868121 (PMC13531343; doi:10.3389/fonc.2026.1868121)
Supplement: Supplementary file 4 [file DataSheet4.docx]

Supplementary Material

**PubMed:**

#1 "Pancreatic Neoplasms"[Mesh] OR ("pancreas cancer"[Title/Abstract] OR "pancreas tumor*"[Title/Abstract] OR "pancreatic cancer"[Title/Abstract] OR "pancreatic carcinoma"[Title/Abstract] OR "pancreatic neoplasm*"[Title/Abstract] OR "pancreatic tumor*"[Title/Abstract])

#2 "Diabetes Mellitus"[Mesh] OR ("diabetes mellitus"[Title/Abstract] OR "diabetes"[Title/Abstract] OR "diabetic"[Title/Abstract] OR "new-onset diabetes"[Title/Abstract] OR "recent-onset diabetes"[Title/Abstract])

#3 "Nomograms"[Mesh] OR "Machine Learning"[Mesh] OR "Deep Learning"[Mesh] OR "Algorithms"[Mesh] OR ("nomogram*"[Title/Abstract] OR "machine learning"[Title/Abstract] OR "deep learning"[Title/Abstract] OR "scoring system*"[Title/Abstract] OR "predictive value"[Title/Abstract] OR "predictive model*"[Title/Abstract] OR "prediction model*"[Title/Abstract] OR "risk model*"[Title/Abstract] OR "risk score*"[Title/Abstract] OR "risk prediction"[Title/Abstract] OR "algorithm*"[Title/Abstract])

#4 #1 AND #2 AND #3

**Embase:**

#1 'pancreas tumor'/exp OR 'pancreas cancer':ab,ti OR 'pancreas tumor*':ab,ti OR 'pancreatic cancer':ab,ti OR 'pancreatic carcinoma':ab,ti OR 'pancreatic neoplasm*':ab,ti OR 'pancreatic tumor*':ab,ti

#2 'diabetes mellitus'/exp OR 'diabetes mellitus':ab,ti OR 'diabetes':ab,ti OR 'diabetic':ab,ti OR 'new-onset diabetes':ab,ti OR 'recent-onset diabetes':ab,ti

#3'nomogram'/exp OR 'machine learning'/exp OR 'deep learning'/exp OR 'algorithm'/exp OR 'predictive value'/exp OR 'nomogram*':ab,ti OR 'machine learning':ab,ti OR 'deep learning':ab,ti OR 'scoring system*':ab,ti OR 'predictive value':ab,ti OR 'predictive model*':ab,ti OR 'prediction model*':ab,ti OR 'risk model*':ab,ti OR 'risk score*':ab,ti OR 'risk prediction':ab,ti OR 'algorithm*':ab,ti

#4 #1 AND #2 AND #3

**Web of Science:**

#1 TS=("pancreas cancer" OR "pancreas tumor*" OR "pancreatic cancer" OR "pancreatic carcinoma" OR "pancreatic neoplasm*" OR "pancreatic tumor*")

#2 TS=("diabetes mellitus" OR "diabetes" OR "diabetic" OR "new-onset diabetes" OR "recent-onset diabetes")

#3 TS=("nomogram*" OR "machine learning" OR "deep learning" OR "scoring system*" OR "predictive value" OR "predictive model*" OR "prediction model*" OR "risk model*" OR "risk score*" OR "risk prediction" OR "algorithm*")

#4 #1 AND #2 AND #3
