# Supplementary material for: Performance of risk prediction models for pancreatic cancer in patients with new-onset diabetes: a systematic review and meta-analysis
Source: Front Oncol. 2026 Aug 18;16:1868121. doi: 10.3389/fonc.2026.1868121 (PMC13531343; doi:10.3389/fonc.2026.1868121)
Supplement: Supplementary file 5 [file DataSheet5.docx]

**Supplementary Table S3. Reporting of calibration and clinical utility measures across included studies.**

| **Study ID** | **Calibration plot** | **Calibration slope** | **Calibration-in-the-large** | **Observed/expected ratio** | **Hosmer-Lemeshow test** | **Decision-curve analysis** | **Risk-score cut-off or risk threshold reported** | **Overall comment** |
| --- | --- | --- | --- | --- | --- | --- | --- | --- |
| **Ali 2024(15)** | **Reported** | **Not reported** | **Not reported** | **Not reported** | **Not reported** | **Not reported** | **Reported** | **Calibration reporting was limited to a calibration plot; a risk threshold was reported.** |
| **Boursi 2017(16)** | **Not reported** | **Not reported** | **Not reported** | **Not reported** | **Reported** | **Not reported** | **Reported** | **Calibration reporting was limited, with only the Hosmer–Lemeshow test reported; risk thresholds were provided.** |
| **Boursi 2022 (IL)(17)** | **Not reported** | **Not reported** | **Not reported** | **Not reported** | **Reported** | **Not reported** | **Reported** | **Calibration reporting was limited to the Hosmer–Lemeshow test; risk thresholds were provided.** |
| **Boursi 2022 (UK)(18)** | **Not reported** | **Not reported** | **Not reported** | **Not reported** | **Not reported** | **Not reported** | **Reported** | **Calibration and clinical utility reporting were limited; a risk-score threshold was reported.** |
| **Chen 2021(19)** | **Not reported** | **Not reported** | **Not reported** | **Not reported** | **Not reported** | **Not reported** | **Reported** | **Calibration and clinical utility reporting were limited; a risk-score threshold was reported.** |
| **Cichosz 2024(20)** | **Not reported** | **Not reported** | **Not reported** | **Not reported** | **Not reported** | **Not reported** | **Reported** | **Calibration metrics were not reported; risk thresholds or risk strata were provided.** |
| **Clift 2024(21)** | **Reported** | **Reported** | **Reported** | **Not reported** | **Not reported** | **Reported** | **Reported** | **Calibration and clinical utility reporting were relatively comprehensive, including calibration plots, quantitative calibration measures, DCA, and risk thresholds.** |
| **Hsieh 2018(22)** | **Not reported** | **Not reported** | **Not reported** | **Not reported** | **Not reported** | **Not reported** | **Not reported** | **Calibration and clinical utility measures were not reported.** |
| **Khan 2021(23)** | **Not reported** | **Not reported** | **Not reported** | **Not reported** | **Not reported** | **Not reported** | **Reported** | **Calibration and clinical utility reporting were limited; an optimal cut-off was reported.** |
| **KhanS 2024(24)** | **Not reported** | **Not reported** | **Not reported** | **Not reported** | **Not reported** | **Not reported** | **Reported** | **Calibration metrics were not reported; risk thresholds were provided.** |
| **Sharma 2018(25)** | **Not reported** | **Not reported** | **Not reported** | **Not reported** | **Not reported** | **Not reported** | **Reported** | **Calibration and clinical utility reporting were limited; a risk-score threshold was reported.** |
| **Sharma 2022(26)** | **Not reported** | **Not reported** | **Not reported** | **Not reported** | **Not reported** | **Not reported** | **Reported** | **Calibration and clinical utility reporting were limited; risk strata were reported.** |
| **Sun 2024(27)** | **Reported** | **Not reported** | **Not reported** | **Not reported** | **Not reported** | **Reported** | **Reported** | **A calibration plot and DCA were reported, but quantitative calibration measures were not provided.** |
| **Zhou 2025(28)** | **Reported** | **Reported** | **Reported** | **Not reported** | **Not reported** | **Reported** | **Reported** | **Calibration and clinical utility reporting were relatively comprehensive, including calibration plot, calibration slope, calibration-in-the-large, DCA, and risk thresholds.** |

Notes: Reported thresholds included model score cut-offs, risk strata, or proposed decision thresholds, but these were not necessarily prospectively validated clinical referral or imaging thresholds. DCA, decision-curve analysis; O/E ratio, observed/expected ratio. “Reported” indicates that the measure was explicitly presented in the original study; “Not reported” indicates that the measure could not be identified from the available publication. Calibration and clinical utility reporting were summarized descriptively and were not pooled quantitatively because of inconsistent reporting formats across studies.
