# Supplementary material for: Performance of risk prediction models for pancreatic cancer in patients with new-onset diabetes: a systematic review and meta-analysis
Source: Front Oncol. 2026 Aug 18;16:1868121. doi: 10.3389/fonc.2026.1868121 (PMC13531343; doi:10.3389/fonc.2026.1868121)
Supplement: Supplementary file 6 [file DataSheet6.docx]

**Supplementary Figure S9. Forest plot of the pooled C-index excluding validation cohorts with imputed variance estimates.**

Sensitivity analysis was restricted to validation cohorts with explicitly reported original confidence intervals or variance measures (k = 10; Refs. [15-16, 18, 20-22, 24, 26-28]), while excluding cohorts that required standard error imputation using the Hanley-McNeil approach (k = 4; Refs. [17, 19, 23, 25]). The pooled C-index among the remaining cohorts was 0.79 (95% CI: 0.76-0.83), which was broadly consistent with the primary pooled estimate of 0.78 (95% CI: 0.75-0.82). Squares represent cohort-specific C-index estimates, horizontal lines represent 95% CIs, diamonds represent random-effects pooled estimates, and square size is proportional to inverse-variance weight. This finding suggests that the overall discrimination estimate was not primarily driven by validation cohorts requiring imputed variance estimates. C-index, concordance index; CI, confidence interval.
